# Proteomic analysis of hypopharyngeal and laryngeal squamous cell carcinoma sheds light on differences in survival

**DOI:** 10.1038/s41598-020-76626-w

**Published:** 2020-11-10

**Authors:** Jiajia Liu, Weiming Zhu, Zhexuan Li, Gengming Cai, Juncheng Wang, Qinglai Tang, Christopher A. Maroun, Gangcai Zhu

**Affiliations:** 1 Department of Otolaryngology-Head and Neck Surgery, The Second Xiangya Hospital, Central South University, Changsha, 410010 China; 2 Fuzhou Medical College of Nanchang University, Fuzhou, 344000 China; 3 Department of Otolaryngology-Head and Neck Surgery, The Xiangya Hospital, Central South University, Changsha, 410008 China; 4 Department of Otolaryngology-Head and Neck Surgery, First Affiliated Hospital of Quanzhou, Fujian Medical University, Quanzhou, 362000 China; 5 Present Address: Department of Otolaryngology-Head and Neck Surgery, Johns Hopkins University, Baltimore, MD 21287 USA

**Keywords:** Immunology, Head and neck cancer

## Abstract

The link between differences in molecular expression and survival among advanced laryngeal (LSCC) and hypopharyngeal squamous carcinoma (HPSCC) remains unclear. Here, we applied the Surveillance, Epidemiology, and End Results (SEER) program, Isobaric tag for relative and absolute quantitation (iTRAQ) with Liquid chromatography-mass spectrometry (LC–MS/MS) proteomics data and The Cancer Genome Atlas (TCGA) related data to discover the possible disparities between HPSCC and LSCC. Our results showed a significantly worse 5-year overall-survival in HPSCC compared with LSCC before and after adjusting for clinical parameters. 240 differentially expressed proteins were enriched in molecular networks of cytoskeleton remodeling and antigen presentation. Moreover, HPSCC consisted of less T-central-memory cells, T-follicular-helper cells, TGF-β response, and CD4^ +^  T memory resting cells, but more wound healing than LSCC. Furthermore, 9 mRNAs expression were  significantly and independently correlated to overall survival in 126 HPSCC and LSCC patients, which was further validated in another cohort of head and neck cancers. These findings support that Immunity signatures as well as pathway networks that include cytoskeleton remodeling and antigen presentation may contribute to the observed differences in survival between HPSCC and LSCC.

## Introduction

Squamous cell carcinoma of the head and neck (HNSCC) represents 95% of head and neck cancer, the sixth lethal cancer, and may affect the oral cavity, oropharynx, hypopharynx, or the larynx^1^. With an annual incidence of more than 500,000 new cases worldwide, HNSCC patients may experience differences in survival due to tumor sub-site origin, clinical stage, metastasis, and HPV status^1,2^. While hypopharyngeal cancers occur in a nearby anatomic location to laryngeal cancers, they usually portend a far worse prognosis^1^. Laryngeal cancers are easily directly inspected by flexible laryngoscopy. However, the examination of the hypopharynx often poses a challenge for clear visualization because of the cramped space and concealed location^3^. As a result, hypopharyngeal cancer is usually diagnosed at a relatively late stage. It is known that cancer patients presenting with late-stage disease usually survive a shorter time than those presenting with earlier-stage disease. Reported overall 5-year survival rates for hypopharyngeal cancer showing a more aggressive course than laryngeal cancer may be biased without taking into consideration of the clinical stage at presentation. Therefore, there is need for a study to compare survival rates in a large population of hypopharyngeal and laryngeal cancer patients with similar clinical characteristics. The Surveillance, Epidemiology, and End Results (SEER) program is well suited for this purpose and provides cancer statistics that is used to better understand the cancer burden amongst the U.S. population. The SEER registry holds clinical information including age, gender, tumor size, neck lymph node, distant metastasis and clinical stage.

In clinical practice, more comprehensive treatment is often recommended by physicians for hypopharyngeal squamous cell cancer (HPSCC) as compared to laryngeal squamous cell cancer (LSCC). Because of the abundant vascular supply and lymphatic drainage, HPSCC is indeed more likely to metastasize than LSCC, even when comparing the same clinical stage^4^. Similar only in histomorphology, there are many pieces of evidence applying immunohistochemistry or RNA sequencing to show several mRNAs (such as SCEL, CRNN, KRT4, SPINK5, and TGM3) and protein(such as Ki67, P53) differences between LSCC and HPSCC^5−8^. One possible contributing factor to the difference in survival between HPSCC and LSCC may be related to these differences in molecular expression^4^. It has been suggested that protein heterogeneity may contribute to the observed differences in survival and likelihood of metastasis between HPSCC and LSCC^4,9–16^. However, other reports indicate that observed differences in survival between HNSCCs from sub-anatomic locations might not reflect differences in molecular profiles between subsites^7,17^. Patient variability in age, clinical stage, tumor size, lymph node status and other factors, as well as limited methodologies (such as comparing a limited number of biomarkers, low-resolution methods, and others) may result in these conflicting conclusions. Therefore, highly sensitive and global protein detection for HPSCC and LSCC patients with similar clinicopathological characteristics is required to provide more thoughtful analyses.

Isobaric tags for relative and absolute quantitation (iTRAQ) can simultaneously mark and quantify global proteins using label peptides, which can be identified by sensitive mass spectrometers^18^. Thus, iTRAQ has significant advantages over some conventional proteomics techniques and is extensively performed with proven value in the discovery of global protein expression^19^. In this study, a proteomic strategy using iTRAQ with liquid chromatography and tandem mass spectrometry (LC–MS/MS) was applied to 10 pairs of cancerous and normal mucosa samples of advanced HPSCC and LSCC patients with similar clinical characteristics. Further analyses based on the Cancer Genome Atlas (TCGA)^20^ datasets support our hypothesis that the significant differentially expressed proteins and their related networks may contribute to the observed difference in survival between hypopharyngeal and laryngeal cancers.

## Results

### Survival differences between hypopharyngeal and laryngeal cancer patients in SEER

After excluding cases with distant metastasis and missing or unknown TNM information, 29,783 of 35,023 cases from the SEER database were included in this study. The clinical characteristics of these patients are summarized in Table 1. Hypopharyngeal and laryngeal cancer groups were significantly different in T and N classification, as well as AJCC clinical stage. Hypopharyngeal cancer patients had a more substantial proportion of T3 or T4 tumors compared to laryngeal cancer patients (52.7% vs. 33.7%, p < 0.001) and a more significant proportion with node-positive disease (67.8% vs. 25.5%, p < 0.001). The majority of hypopharyngeal cancer patients presented with stage IV disease (61.3%) compared to only 25.3% in the laryngeal cancer group, while only 4.9% of the hypopharyngeal cancer patients presented with stage I compared to 38.9% in the laryngeal group (p < 0.001).

**Table 1:** Characteristics of hypopharyngeal cancer and laryngeal cancer Patients from the SEER Database.

	Unmatched cases			Matched cases		
	Hypopharynx	Larynx	*p*	Hypopharynx	Larynx	*p*
Total cases	4256	25,527	0.317	4256	4256	0.642
Age (χ ± SD (years))	64.79 ± 11.02	64.99 ± 11.50		64.79 ± 11.02	64.70 ± 10.79	
**Gender (%)**						
Male	3425 (80.4%)	20,560 (80.5%)	0.822	3421 (80.4%)	3436 (80.7%)	0.701
Female	835(19.6%)	4967(19.5%)		835(19.6%)	820(19.3%)	
**T classification (%)**						
T1-2	2009(47.3%)	16,912(56.3%)	< 0.001	2009(47.3%)	2022(47.5%)	0.794
T3-4	2247 (52.7%)	8615 (33.7%)		2247 (52.7%)	2234 (52.5%)	
**N classification (%)**						
N−	1373(33.2%)	19,011(74.5%)	< 0.001	1373(33.3%)	1374(33.3%)	1
N+	2887 (67.8%)	6516 (25.5%)		2883 (67.7%)	2882 (67.7%)	
**AJCC clinical stage (%)**						
I	207 (4.9%)	9921 (38.9%)	< 0.001	207 (4.9%)	207 (4.9%)	1
II	546 (12.8%)	4488 (17.6%)		546 (12.8%)	546 (12.8%)	
III	894 (21.0%)	4635 (18.2%)		894 (21.0%)	891 (20.9%)	
IV	2609 (61.3%)	6483 (25.3%)		2609 (61.3%)	2612 (61.4%)	

Note: χ ± SD means mean ± standard deviation; p < 0.001 defined as significant; N + means clinical lymph node metastasis; N0 means without neck lymph node metastasis, the percentage was calculated on number of which divided by total cases.

**Figure 1:**
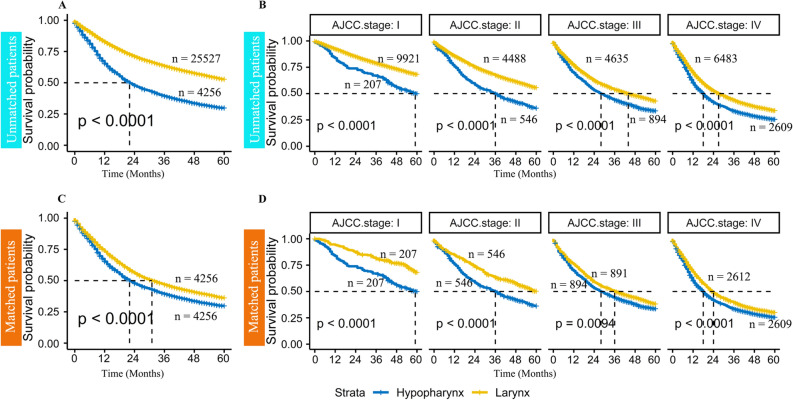
Comparison of overall survival (OS) hypopharyngeal and laryngeal cancer patients from the SEER database (**A**) Kaplan–Meier analysis in 29,793 patients with hypopharyngeal or laryngeal cancer showed that hypopharyngeal cancer patients have poorer overall survival than laryngeal cancer patients (median survival time: 22 months vs. greater than 60 months, p < 0.01); (**B**) Kaplan–Meier analysis showing difference in survival between hypopharyngeal and pharyngeal cancer independent of AJCC clinical-stage (all p < 0.01); (**C**) After adjusting for age, gender, T classification, N classification, and AJCC clinical-stage, Kaplan–Meier analysis in 4256 pairs of hypopharyngeal and laryngeal cancer patients continue to show poorer survival in hypopharyngeal cancer patients (median survival time: 22 months vs. 34 months, p < 0.01); (**D**) Kaplan–Meier analysis in matched patients with hypopharyngeal or laryngeal cancer showing hypopharyngeal cancer patients with worse prognosis in early and late clinical-stage (all p < 0.01).

It was thought that any difference in survival of patients with hypopharyngeal or laryngeal cancer might be confounded by the differences in clinical characteristics, as shown in Table 1. Therefore, propensity score matching was applied to match hypopharyngeal and laryngeal cancer patients to get rid of biases from age, gender, T classification, N classification, and AJCC clinical stage (Table 1). After adjustment of these clinical parameters, Kaplan–Meier survival analysis remained to show that patients with hypopharyngeal cancer have worse survival than those with laryngeal cancer (Fig. 1, median survival time: 22 months vs. 34 months). Furthermore, when stratified by clinical stage, it is shown that hypopharyngeal cancer patients have significantly worse overall survival regardless of the clinical-stage (Fig. 1, Stage I-IV p < 0.001). The same finding was also observed when stratified by T stage (Supplementary Fig. 1).

### Clinical characteristics of the iTRAQ-proteomics cohort

As the majority of hypopharyngeal cancer patients included in our SEER analysis were stage III-IV, we included only advanced stage HPSCC and LSCC patients in our proteomics cohort. There were 10 HPSCC and 10 LSCC tumor-normal pairs included; clinical characteristics are summarized in Table 2. All patients had node-positive disease, and none had metastatic disease at presentation. The pathological grade was similar between the two cohorts. All included patients were males with a history of smoking.

**Table 2:** Clinical parameters of 10 pairs of advanced HPSCC and LSCC patients.

Parameter	HPSCC (patients)	LSCC (patients)
**Clinical stage**		
I–II	0	0
III–IV	10	10
**T classification**		
T1–T2	4	1
T3–T4	6	9
**N classification**		
N0	0	0
N+	10	10
**M classification**		
M0	10	10
M+	0	0
**Pathological grade**		
Low	0	0
Middle	4	4
High	6	6
**Sex**		
Male	10	10
Female	0	0
**Age**		
≤ 55 years	5	3
> 55 years	5	7
**Smoking**		
Yes	10	10
No	0	0

Note: N + means clinical lymph node metastasis, N0 means without neck lymph node metastasis, 55 years is the median age of HPSCC and LSCC in our hospital clinical dataset.

### The relative expression of global proteins in HPSCC and LSCC patients

A differential protein profile between hypopharyngeal (HPM) and laryngeal (LM) normal mucosa was first examined to establish a reference for baseline differences and to account for variation between individual samples. An abundance of 613 proteins was found to be altered in HPM compared to LM, with 515 proteins being overexpressed, and 98 proteins under expressed (Table S1, Supplementary Data). Thirty proteins did not show differential expression in HPM vs. LM (Table S2, Supplementary Data).

424 proteins were founded to be differentially expressed in HPSCC vs. LSCC samples. To account for the baseline variations found in the analysis of normal mucosa samples, 184 differentially expressed proteins that were common to both tumor and normal mucosa analyses were excluded from the 424-protein list. The remaining 240 proteins were considered tumor-related differentially expressed proteins and included Vimentin, β-catenin, HLA-A/B/C, and MICA, among others (Table S3, Supplementary Data).

### GO Enrichment Maps and Networks for significantly altered proteins

**Figure 2:**
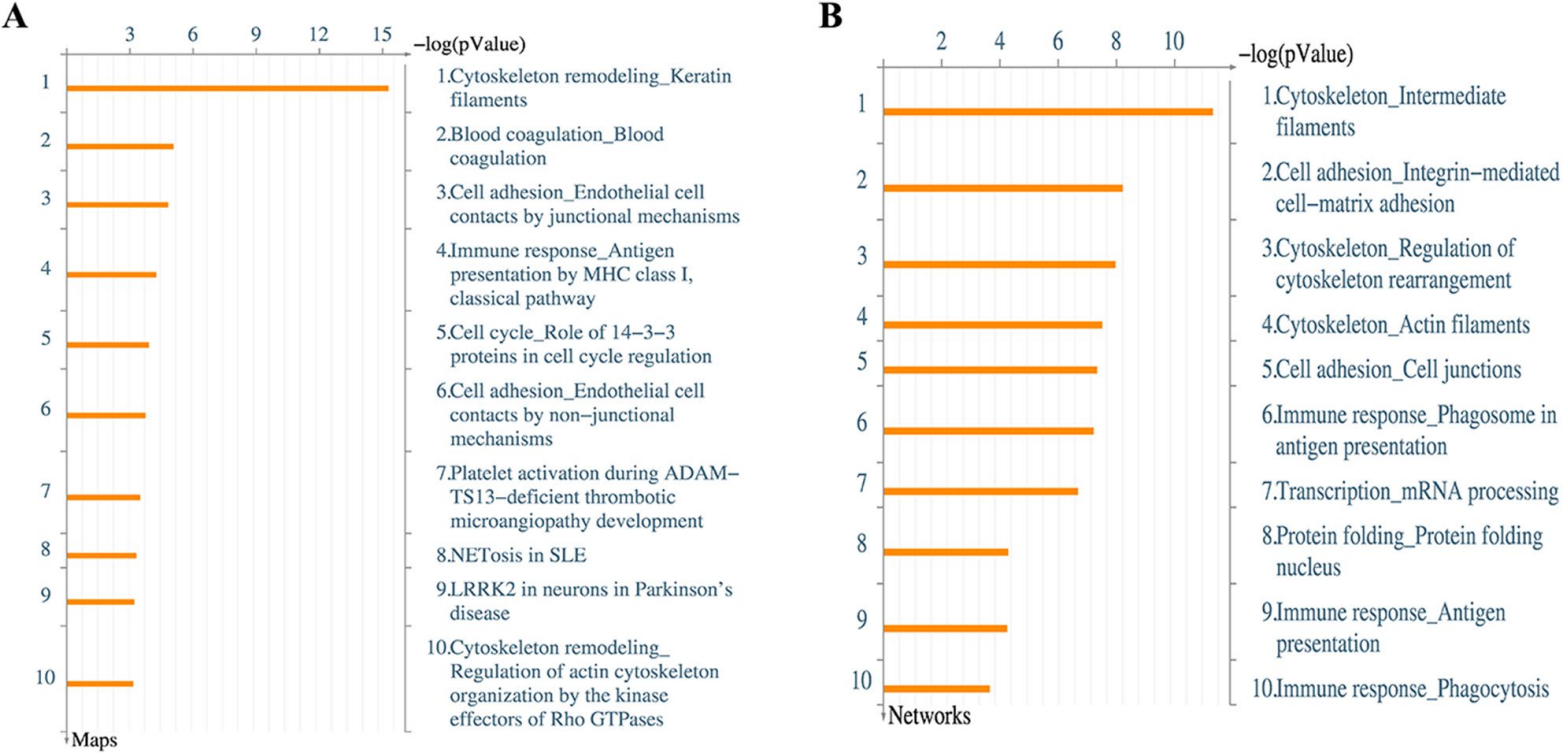
Gene ontology enrichment maps for significantly altered proteins. (**A**,**B**) Top 10 biological networks and maps of significantly expressed proteins in HPSCC vs. LSCC. X-axis indicates the confidence of pathway enrichment; Y-axis shows the rank of the enrichment pathway.

**Figure 3:**
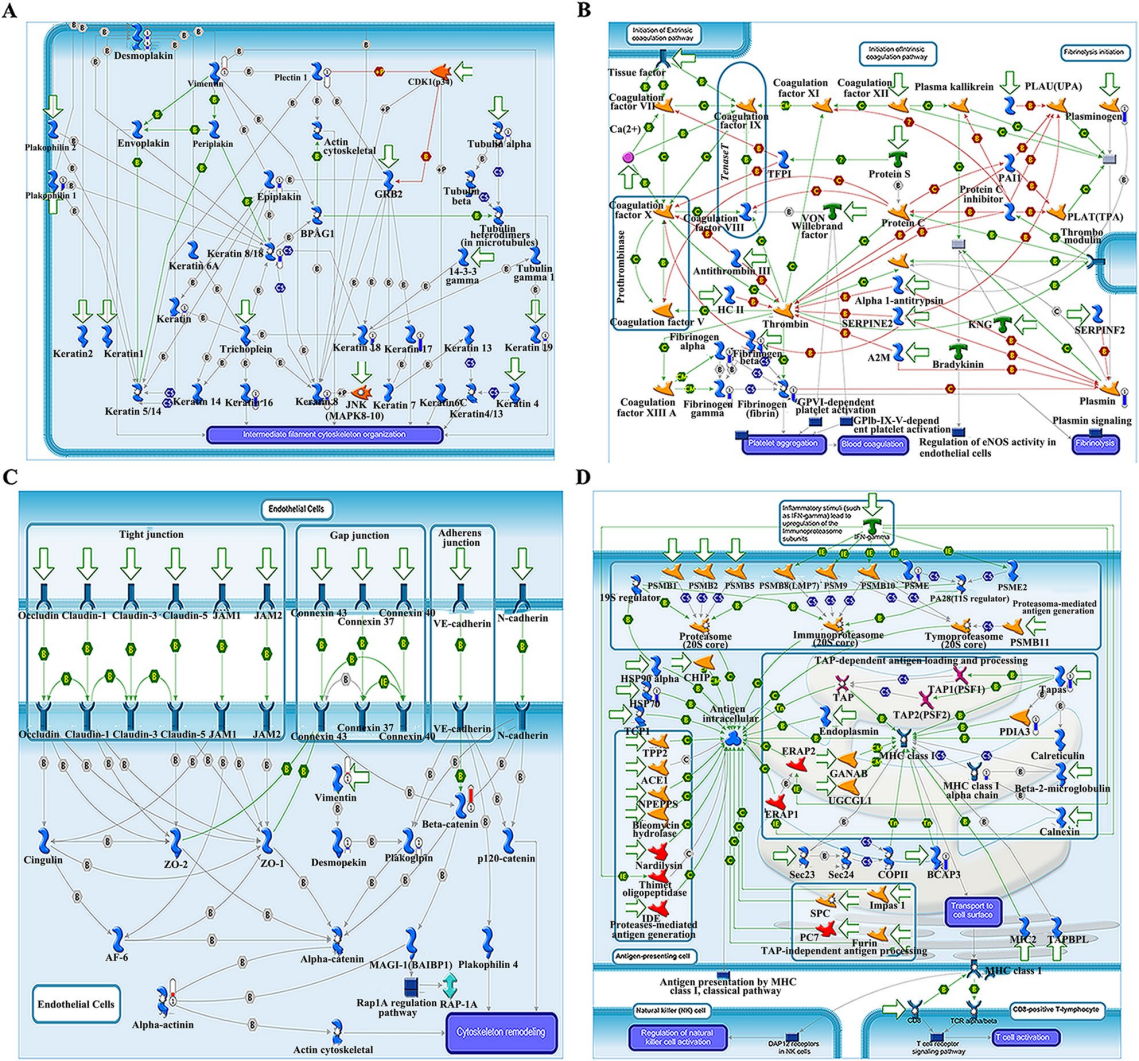
Molecular networks for significantly altered proteins by Metacore analysis. Enriched pathways consisting of 240 significant proteins are visualized on maps of cytoskeleton organization (**A**,**C**), platelet aggregation (**B**), and antigen presentation (**D**). Upward red indicate up-regulated signals, and downward (blue) ones indicate down-regulated gene expression levels.

To annotate the above 240 differentially expressed proteins, we applied the MetaCore mapping tool to generate an enrichment analysis of pathway maps and cellular processes (Fig. 2A-B, respectively). MetaCore has the ability of generating both pathway maps, which are manually created based on known canonical pathway data, as well as networks, which are automatically created by the tool based on existing protein interactions within a database. The Gene Ontology analysis indicated that the most significantly enriched pathway networks were involved in cytoskeleton remodeling/keratin filaments, cell adhesion/endothelial cell contacts by junctional mechanisms, and immune response/antigen presentation by MHC class I pathway (Fig. 3A–D).

### The landscape of cancer immunity in hypopharyngeal and laryngeal cancer

**Figure 4:**
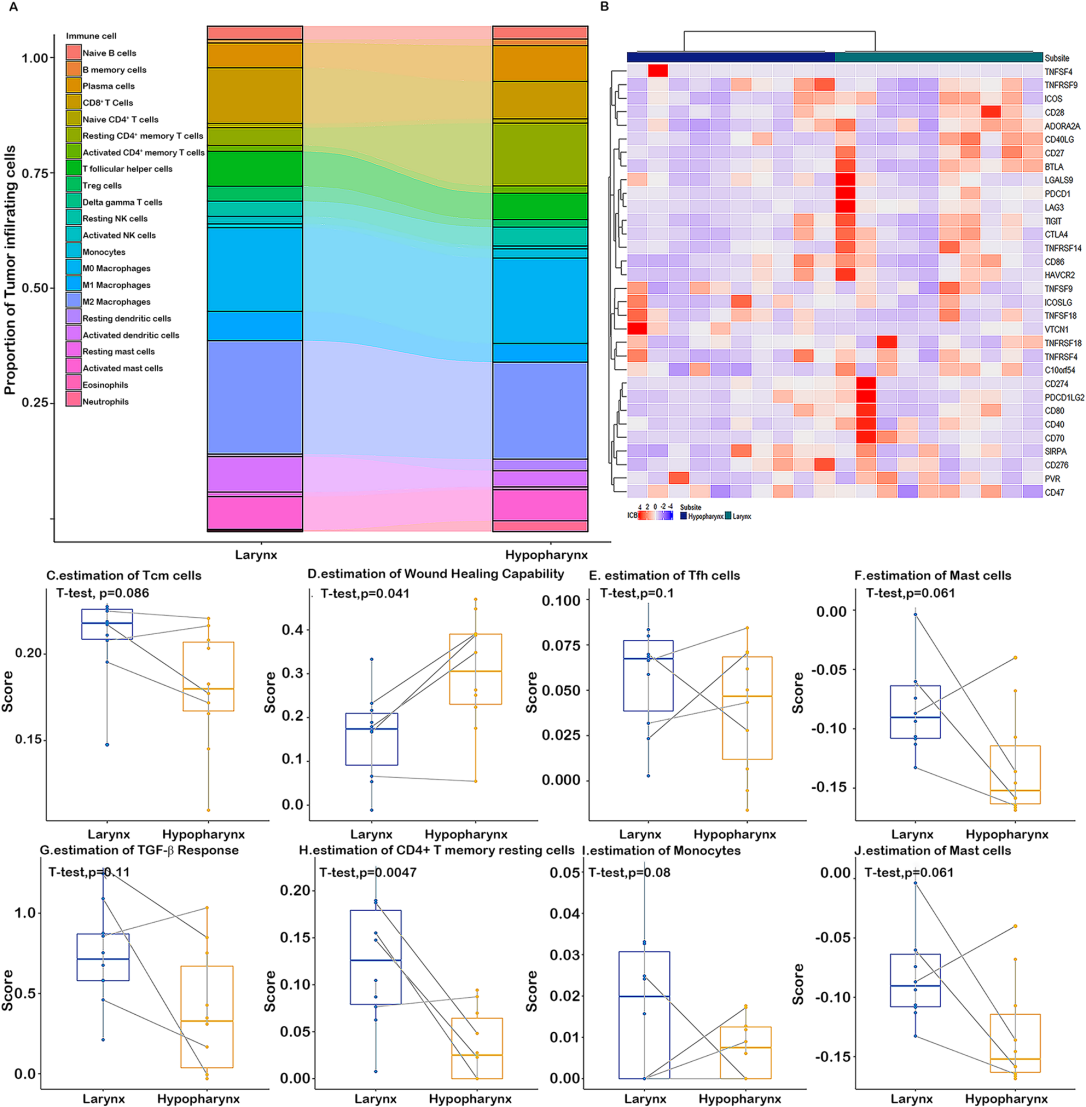
The landscape of cancer immunity in hypopharyngeal and laryngeal cancer. (**A**) P of cancer infiltrating immune cells in clinically similar hypopharyngeal and laryngeal cancers. (**B**) Scaled expression of known immune checkpoint biomarkers on the cell surface of clinically similar hypopharyngeal and laryngeal cancers. (**C**–**J**) Significantly different cell signatures in HPSCC and LSCC patients. Paired student t-test analysis shows significant differences in Tcm cells, Tfh cells, TGF-beta response, CD4^ +^  T memory resting cells, and wound healing capability between 10 matched hypopharyngeal and laryngeal cancer patients (all p < 0.05).

The proportion of cancer infiltrating immune cells was deconvoluted by CIBERSORT method in hypopharyngeal and laryngeal cancer patients with matched clinical stage, gender and age from the TCGA public dataset^21,22^(Fig. 4A). The proportions of CD4^ +^  T memory resting cells and mast cells appeared to be drastically different between laryngeal and hypopharyngeal cancer. Moreover, we constructed a heatmap for known immune checkpoint biomarkers (Fig. 4B), which indicated the differential expression of immune checkpoints such as CTLA-4, LAG-3 and TIGIT.

Considering that the significantly expressed proteins in the above iTRAQ results were enriched in the cytoskeleton remodeling and immune response pathways, immune infiltration and wound healing were further compared in 10 paired hypopharyngeal and laryngeal cancer patients (Table S4, Supplementary Data). As shown in Fig. 4C–J, hypopharyngeal cancer patients had less T central memory (Tcm) cells, T follicular helper (Tfh) cells, TGF-beta response, and CD4^ +  ^T memory resting cells, but a higher wound healing score than laryngeal cancer patients (p < 0.05).

### mRNA expression and correlation with survival in hypopharyngeal and laryngeal cancer

**Figure 5:**
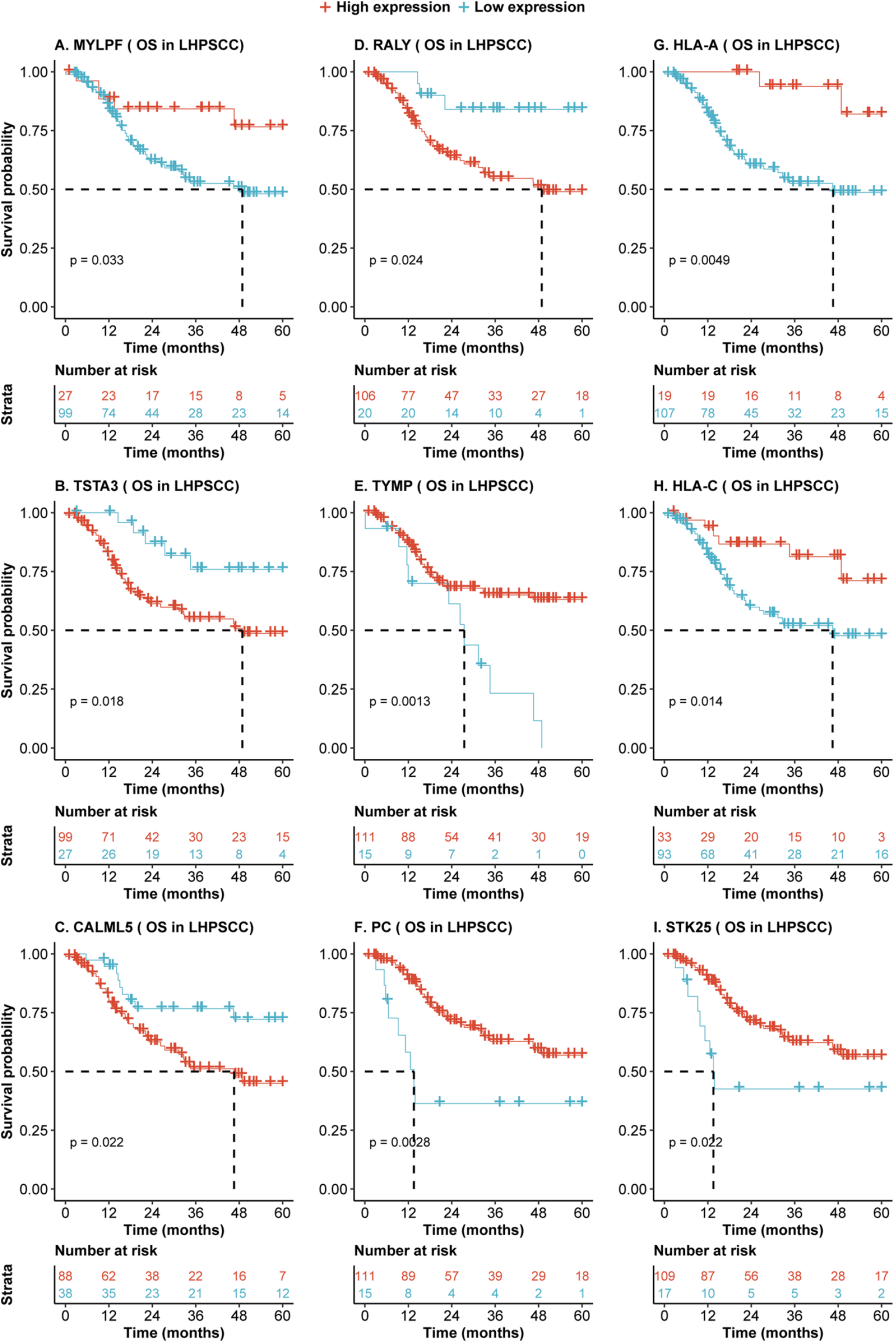
Aberrant expression of mRNAs predicts different overall survival of 126 HPSCC and LSCC patients. (**A**–**I**) Kaplan–Meier analysis shows that high mRNA expression of *MYLPF*, *HLA-A/-C, TYMP, PC,* and *STK25* and low expression of *CALML5, TSTA3,* and *RALY* are associated with better overall survival time in 126 HNSCC patients (all p < 0.05). Dotted line represents median survival time. Red = high expression group, light blue = low expression group.

**Figure 6:**
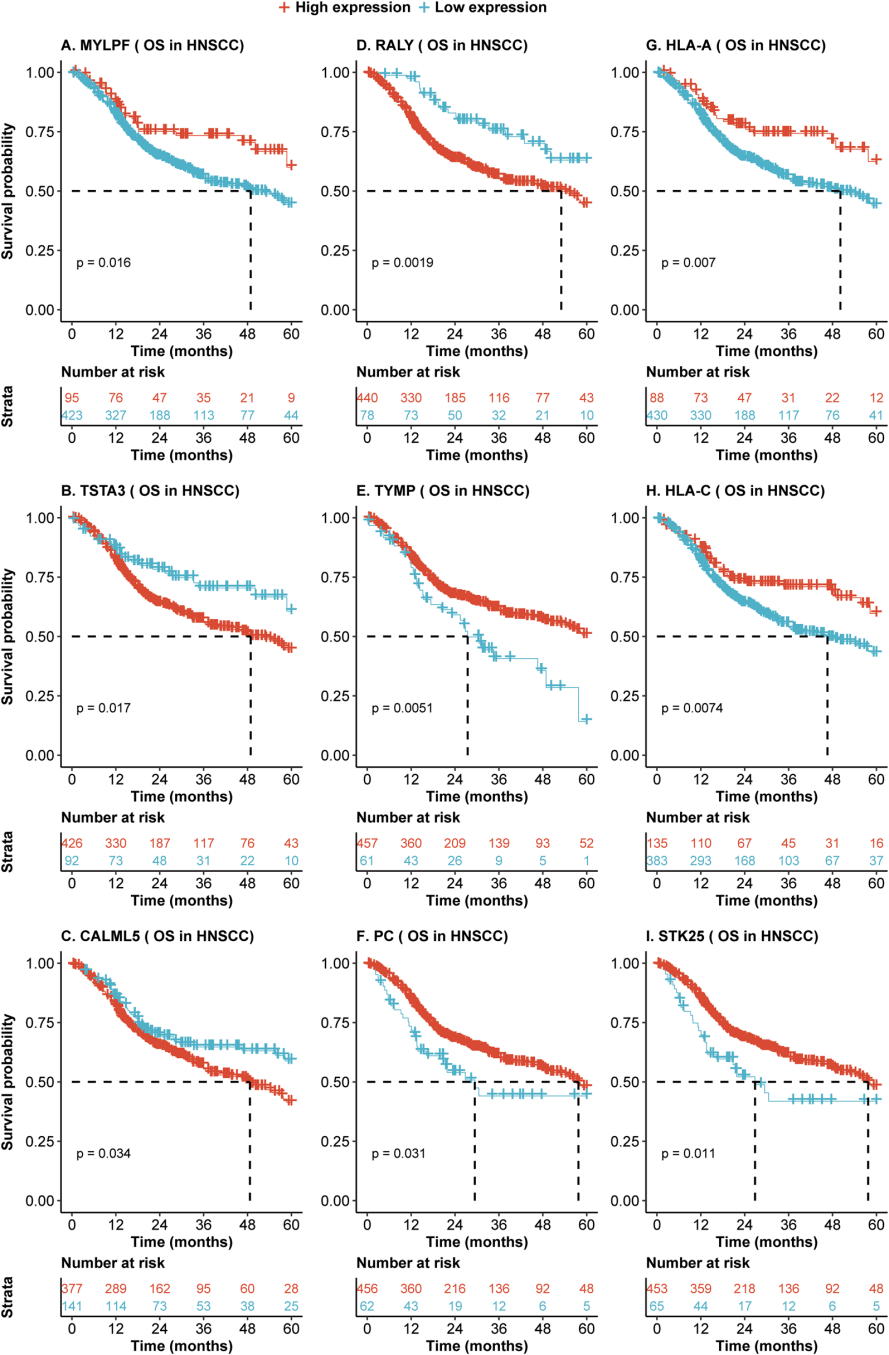
Aberrant mRNA expression predicts different overall survival of 513 HNSCC patients. (**A**–**I**) Kaplan–Meier analysis showed that high mRNA expression of *MYLPF*, *HLA-A/-C, TYMP, PC,* and *STK25* and low expression of *CALML5, TSTA3,* and *RALY* are associated with better overall survival time in 513 HNSCC patients (all p < 0.05). Dotted line represents median survival time. Red = high expression group, light blue = low expression group.

To investigate whether the differentially expressed proteins may contribute to differences in prognosis in hypopharyngeal cancer and laryngeal cancer, we analyzed the mRNAs corresponding to these 240 significantly expressed proteins in hypopharyngeal and laryngeal cancers derived from the TCGA dataset. On univariate Cox regression analysis, mRNA expression of 53 genes was significantly associated with overall survival in 126 hypopharyngeal cancer and laryngeal cancer patients (Table 3). Moreover, Kaplan–Meier analysis indicated that high or low expression of genes, including *RALY, TSTA3, and HLA-A*, was associated with significant differences in survival in hypopharyngeal cancer and laryngeal cancer patients (p < 0.05, Fig. 5). To validate whether these findings could extend to other head and neck cancers, univariate and multivariate Cox proportional hazards regression analysis was performed to validate correlations of the 53 genes and overall survival outcomes in 519 HNSCC patients across all subsites, including the oral cavity and oropharynx. 9 of 53 genes were confirmed in the 519 HNSCC patients. The Hazard ratios and p values across the cancer groups are summarized in Table 3, and the associated survival curves were visualized and compared by Kaplan–Meier analysis (Fig. 6).

**Table 3:** Significant correlations between mRNAs and overall survival time of LHPSCC and its validation in HNSC patients.

mRNA	126 LHPSCC patients				Validation dataset (519 HNSCC patients)			
	Univariate HR (95% CI)	*P* value	Multivariate* HR (95% CI)	*P* value	Univariate HR (95% CI)	*P* value	Multivariate* HR (95% CI)	*P* value
MYLPF	2.6 (1.04–6.70)	0.04	3.11 (1.08–8.94)	0.04	1.7 (1.10–2.53)	0.02	1.55 (1.01–2.39)	0.05
TSTA3	0.34 (0.14–0.87)	0.02	0.31 (0.11–0.83)	0.02	0.6 (0.39–0.92)	0.02	0.63 (0.41–0.96)	0.03
CALML5	0.43 (0.21–0.90)	0.03	0.37 (0.16–0.86)	0.02	0.7 (0.51–0.98)	0.04	0.62 (0.44–0.87)	0.01
RALY	0.28 (0.09–0.91)	0.04	0.26 (0.08–0.87)	0.03	0.49 (0.31–0.78)	0.00	0.47 (0.3–0.75)	< 0.01
TYMP	2.9 (1.47–5.78)	< 0.01	2.48 (1.15–5.33)	0.02	1.7 (1.17–2.50)	0.01	1.79 (1.21–2.65)	< 0.01
PC	2.9(1.40–6.06)	< 0.01	3.13 (1.4–7.02)	0.01	1.5 (1.04–2.28)	0.03	1.51 (1.01–2.27)	0.04
HLA_A	6 .0(1.45–24.76)	0.01	6.75 (1.58–28.78)	0.01	1.8 (1.17–2.79)	0.01	1.83 (1.17–2.86)	0.01
HLA_C	2.8 (1.19–6.62)	0.02	5.72 (2.05–15.91)	< 0.01	1.6 (1.13–2.297)	0.01	1.69 (1.17–2.45)	0.01
STK25	2.3 (1.11–4.79)	0.03	2.96 (1.3–6.74)	0.01	1.6 (1.11–2.40)	0.01	1.63 (1.1–2.41)	0.02

HR: hazard ratio of death for patients with low expression of mRNA over its high expression; OS: Overall survival, CI: confidence interval, p value less than 0.05 was considered as significant. LHPSCC: laryngeal and hypopharyngeal squamous cell carcinoma; HNSCC: Head and neck squamous cell carcinoma; * HR and p value were adjusted age, gender, race, T/N/M stage and clinical stage in the Cox proportional hazards regression analysis.

## Discussion

HNSCC is represented by a heterogeneous group of cancers in various anatomic locations^1,23^. A recent EUROCARE population-based study indicated that the 5-year relative survival rate was lowest for hypopharyngeal cancer (25%) and highest for laryngeal cancer (59%)^24^, which is consistent with our findings. Most interestingly, these drastically different survival rates are attributed to cancers that arise in such geographically close anatomic locations^24,25^. As is known, some clinical parameters, such as clinical stage, age, and gender, may influence the survival time of cancer patients^26^. One argument as to why patients with hypopharyngeal cancer often do worse than laryngeal cancer patients is that hypopharyngeal cancer is usually diagnosed at a more advanced stage. Only 4.9% of the hypopharyngeal cancer patients presented with stage I cancer, compared to 38.9% in the laryngeal group in this study. However, using propensity score matching, we compared the survival of hypopharyngeal and laryngeal cancer with similar clinical parameters, including clinical stage, T stage, age, gender, and neck node status. Our findings demonstrate that even amongst clinical stage I and T1-2 tumors, hypopharyngeal cancer portends a worse prognosis. Our study suggests that HPSCC may demonstrate more aggressive biology irrespective of age, gender, T classification, N classification, and AJCC clinical stage. To our best knowledge, this is the first study to compare the survival of HPSCC and LSCC controlling for these clinical factors in a large population. Another explanation for the worse survival in HPSCC is the abundant vascular supply and lymphatic drainage in HPSCC. However, the oropharynx is very similar to the hypopharynx in terms of its tissue structure, and also boasts a robust vascular supply with ample lymphatic tissue. Despite this, oropharyngeal squamous cell carcinoma patients tend to have better survival than HPSCC patients. Therefore, the anatomic structure alone in HPSCC does not sufficiently explain the higher likelihood of a worse prognosis. As such, we turned our attention towards a molecular characterization of HPSCC to investigate its possible contribution to its worse outcomes.

There have been several studies examining the transcriptomic and genomic heterogeneity in HNSCC across various subsites, including oral cavity, tonsil, and oropharynx^27−29^. These works showed controversial conclusions in comparing the genomes and their expression^7,17^. However, there have been no studies for the specific comparison between hypopharyngeal and laryngeal cancer. In this work, we employed iTRAQ (2D) LC–MS/MS proteomic analysis to measure the global protein expression in HPSCC and LSCC. Traditional methods for detecting protein expression, such as western blotting, usually limits the number of proteins that can be investigated simultaneously. However, our proteomic method can yield information on hundreds of proteins from a relatively small amount of tissue. Furthermore, iTRAQ has the ability to cover more peptides and provides sensitive quantification compared with traditional methods, like DIGE^30^.

Although this study had a limited number of sample pairs for iTRAQ proteomic analysis, we employed a strict method of analysis to increase the confidence in our results. To eliminate intra-patient variation, normal mucosa from the same patients were simultaneously analyzed with tumor tissues. Additionally, our proteomic samples from HPSCC and LSCC were selected based on having similar clinical parameters like clinical stage, to decrease confounding bias. Two hundred forty differentially expressed proteins and 208 non-altered proteins were found in the comparison of HPSCC and LSCC, which indicates that both molecular differences and similarities exist. The 208 similarities may represent histological and anatomical commonalities between HPSCC and LSCC. Regarding the differences, it would be both interesting and clinically meaningful if the observed differences in survival are related or even explained in part by the distinct protein expression patterns seen.

The differentially expressed proteins that we identified were analyzed using Metacore, a precise, comprehensive pathway analysis and knowledge mining tool that delivers high-quality biological systems content in context^31^. Cytoskeleton remodeling (intermediate filaments, integrin-mediated cell–matrix adhesion, and cytoskeleton rearrangement) and phagosomes in antigen presentation were the top networks enriched by these 240 proteins. Cytoskeleton remodeling in the cancer cell has been linked with increased cell mobility and facilitated metastasis, which may be linked to unfavorable survival time in some patients^32^. Strikingly, we found that many of these proteins were enriched in pathways related to antigen presentation by MHC Class I and phagosomal machinery. In consideration of the crucial effect of antigen presentation on immune-mediated cancer clearance, this data suggests the need for further investigation into the role of innate or adaptive immunity-related differences in HPSCC and LSCC.

TCGA is a public landmark cancer genomics program with the aim to catalog and discover major genomic alterations to create a comprehensive “atlas” of 11,000 primary cancers^33^. The immunity signatures using mRNA expression data from the TCGA was obtained from previously published work^21,22^. Here, we showed that hypopharyngeal cancer had less T central memory cells, T follicular helper cells, TGF-beta response, and CD4 ^+^ T memory resting cells, but a higher wound healing score than laryngeal cancer. These findings suggest that hypopharyngeal cancers display an altered immune response that may potentially affect the survival seen compared with laryngeal cancer. In addition, taken together with the differentially expressed immune-checkpoint related proteins found between HPSCC and LSCC, these findings may have clinical implications with regards to response to checkpoint inhibition for these cancers in clinical trials^34^.

We also found that expression of 53 out of 240 genes was significantly associated with overall survival on univariate logical regression analysis in 126 HPSCC and LSCC patients using the TCGA dataset. Similar correlations of survival with 9 of these 53 genes were observed when including all 519 HNSCC patients in the TCGA cohort across all subsites, including the oral cavity and oropharynx (Fig. 6). This data provides support that the observed survival differences among HPSCC and LSCC patients may be related to differential expression of these 9 proteins, including RALY, TSTA3, and HLA-A. RALY, a member of the heterogeneous nuclear ribonucleoprotein (hnRNP), is thought to be involved in mRNA splicing and metabolism. Its role in tumorigenesis and development remains unclear. However, it has been reported as an oncogene in hepatocellular carcinoma^35^, clear cell renal cell carcinoma^36^, triple-negative breast cancer^37^, and non-small-cell lung cancer^38^. TSTA3, also known as GFUS, participates in the pathway of transporting to the Golgi apparatus as well as metabolism^39^, and is considered an oncogene in many cancers including esophageal squamous cell carcinoma^40^. To the best of our knowledge, this is the first study to report the correlation of RALY and TSTA3 with overall survival in head and neck cancer. Finally, high expression of HLA-A or HLA-C is a favorable factor for head and neck cancer patients, which is as this correlates with increased antigen presentation leading to immune-mediated tumor clearance.

There are several limitations in our study. While a main advantage of our study is our selection of patients based on similar clinical characteristics in order to minimize the effect of these as confounding factors, we aren’t able to eliminate any bias that may arise from the development of the individual tumors themselves. Specifically, without fully characterizing the timeline of these protein aberrations with regard to tumor development, it is not understood whether these differences in protein expression are a cause or a result of tumor development itself. Additionally, while we performed our proteomic analysis using a small sample of patients from our own institution, we performed our experimental validation using mRNA expression data from the TCGA. While mRNA transcript and protein levels often covary closely within the cell, one is not a perfect surrogate for the other. Ideally, future experiments using the same proteomic analysis in larger cohort should be performed in order to validate our findings. Additionally, in vivo experiments, such as gene knockout experiments in mice may be more informative regarding the clinical implications of the genes discovered in this exploratory analysis. Finally, an important limitation in our study is the lack of information regarding type of treatment patients received. It is plausible that some bias is introduced, especially with regards to survival outcomes, related to whether a patient received surgery, chemotherapy, radiation, or any combination of these. Unfortunately, a majority of the patients included in our study had treatment information that was either missing or incomplete. In this setting, the inclusion of treatment as a covariate would create additional bias from the exclusion of such a large percentage of our sample population, and would significantly reduce the power of our study. By controlling for cancer stage, however, it was thought that the risk of bias from treatment effect would be somewhat minimized, as patients with similar stage, especially later stage, are more likely to undergo a similar course of treatment with multimodality therapy.

## Conclusion

We showed hypopharyngeal carcinoma patients survived significantly poorer than laryngeal carcinoma independent of age, gender, tumor size, neck lymph node status, and AJCC clinical stage. There are 240 proteins differently expressed in hypopharyngeal carcinoma and laryngeal carcinoma, which are enriched in the networks of cytoskeleton remodel and antigen presentation. Nine of the above 240 molecules correlated to the overall survival time in HPSCC/LSCC and HNSCC. Hypopharyngeal carcinoma had less Tcm cells, Tfh cells, TGF-β response, and CD4 ^+  ^T memory resting cells, but more wound healing than laryngeal carcinoma.

In addition to diagnosed at relatively late clinical stage abundant, vascular supply and lymphatic drainage, our data may have implications that differential expressed proteins may be some of reasons for hypopharyngeal cancer has poorer prognosis as compared to laryngeal cancer. This study provides a comprehensive view about the disparities between HPSCC and LSCC, which remind us these differences when treating patients or designing clinical trials.

## Materials and methods

### Data sources

We obey the principles of the 1983 Declaration of Helsinki. All of experiments in this paper obey this principle. Informed consent was obtained from all subjects or, if subjects are under 18, the informed consent of a parent and/or legal guardian was obtained. Informed consent was obtained from all patients before undergoing surgery, and all experiments were conducted by following the bioethics rules issued by the Research Ethics Committee of Central South University, Changsha, China. The Surveillance, Epidemiology and End Results (SEER) program provides statistical information about the cancer burden amongst the U.S. population. Clinical variables collected in SEER include age, gender, tumor size, lymph node metastasis, distant metastasis, and clinical stage^41^. The SEER database was queried for case-based data from 2004 to 2013, using the SEER 18 Registry Research Data plus Hurricane Katrina Impacted Louisiana Cases, Nov 2018 (1975–2016 varying) submission. Five years was considered to be the endpoint of follow-up. Site recode ICD-0–3/WHO 2008″ was filtered by “Hypopharynx” or “Larynx”. Cases with distant metastasis or with missing or unknown information regarding TNM, gender, age, or clinical stage were excluded from the analysis. HNSCC normalized mRNA expression and corresponding clinical data for the TCGA cohort were acquired from the Broad Institute GDAC Firehose browser interface (https://gdac.broadinstitute.org/) and TCGA-CDR-Paper^42^. Protein data were utilized from our previously published iTRAQ (2D) LC–MS/MS work^43,44^.

### iTRAQ proteomic analysis

The iTRAQ (2D) LC–MS/MS experiments were performed previously^43,44^. Briefly, normal mucosa and primary tumor samples of 10 HPSCC and 10 LSCC patients were collected under the approval of the Medical Research Ethics Committee of Xiangya Hospital (Changsha, China). The hypopharyngeal normal mucosa and primary tumor specimens were labeled IT118 and IT121, while the laryngeal normal and tumor specimens were labeled IT115 and IT113, respectively. The relative global protein expression in HPSCC and LSCC samples was analyzed using Protein Pilot v3.0 software (Applied Biosystems) according to the human International Protein Index (IPI) database v3.45.

To minimize the false positive rate, a strict cutoff for protein identification was used based on the following criteria: unused ProtScore > 1.3 and more than one peptide with 95% confidence per repetition. It was shown that 43,673 spectra, 19,882 peptides, and 853 proteins were identified and quantified by the calibration with a 5% global false discovery rate. Protein relative expression ratios were based on the peak area ratios of the peptides from the same protein. The resulting dataset was auto bias-corrected to eliminate any variability due to the unequal mixing of the variously labeled digests. A fold change in protein expression greater than 1.2 or less than 0.8 was considered significant, with values in between considered as similar expression. MetaCore (Gene-Go; St. Joseph, MI, USA) from Clarivate Analytics, an integrated program with manual databases and practical algorithms for functional analysis, was applied to annotate the functions of the differentially expressed proteins ^45^.

### Software and statistics

All statistical analyses were performed using Rstudio (version 3.5.3, https://cran.r-project.org/). Briefly, Kaplan–Meier survival analysis was performed using R packages (‘survival’, ‘survminer’). The “surv_cutpoint” command was used to identify the best cutoff for ‘High expression’ or ‘Low expression’ in Kaplan–Meier survival analysis.

The log-rank test was used to analyze differences in survival, with a p-value of less than 0.05 considered statistically significant. Overall survival was censored at a maximum time of 60 months. Univariable Cox proportional hazards regression models were used to correlate survival with mRNA expression. The violation of the proportional hazards assumption was tested using the ‘cox.zph’ function in the "survminer" package. Propensity score matching (PSM) was performed for matching clinical parameters for patients in SEER and TCGA datasets by R package “MatchIt”^46^.

## Supplementary information

Supplementary information.
