# Supplementary material for: Proteomic analysis of hypopharyngeal and laryngeal squamous cell carcinoma sheds light on differences in survival
Source: Sci Rep. 2020 Nov 10;10:19459. doi: 10.1038/s41598-020-76626-w (PMC7655956; doi:10.1038/s41598-020-76626-w)
Supplement: Supplementary file 1 — Supplementary information. [file 41598_2020_76626_MOESM1_ESM.pdf]

Title:

Proteomic analysis of hypopharyngeal and laryngeal squamous cell carcinoma sheds light on differences in survival

Running title: Proteomics and survival in HPSCC and LSCC

Authors: Jiajia Liu[1, *], Weiming Zhu[2, *], Zhexuan Li[3, *], Gengming Cai[4], Juncheng Wang[3], Qinglai Tang[1], Christopher A Maroun[5], Gangcai Zhu[1,5,✉]

Affiliations:

[1] Department of Otolaryngology-Head and Neck Surgery, The Second Xiangya Hospital, Central South University, Changsha 410010, China.

[2] Fuzhou Medical College of Nanchang University, Fuzhou 344000, China.

[3] Department of Otolaryngology-Head and Neck Surgery, The Xiangya Hospital, Central South University, Changsha 410008, China.

[4] Department of Otolaryngology-Head and Neck Surgery, First Affiliated Hospital of Quanzhou, Fujian Medical University, Quanzhou, 362000, China.

[5] Current address: Department of Otolaryngology-Head and Neck Surgery, Johns Hopkins University, Baltimore, Maryland 21287, U.S.A.

[*] Contribute equally

[✉] Corresponding author: Gangcai Zhu, E-mail: qianhudoctor@csu.edu.cn

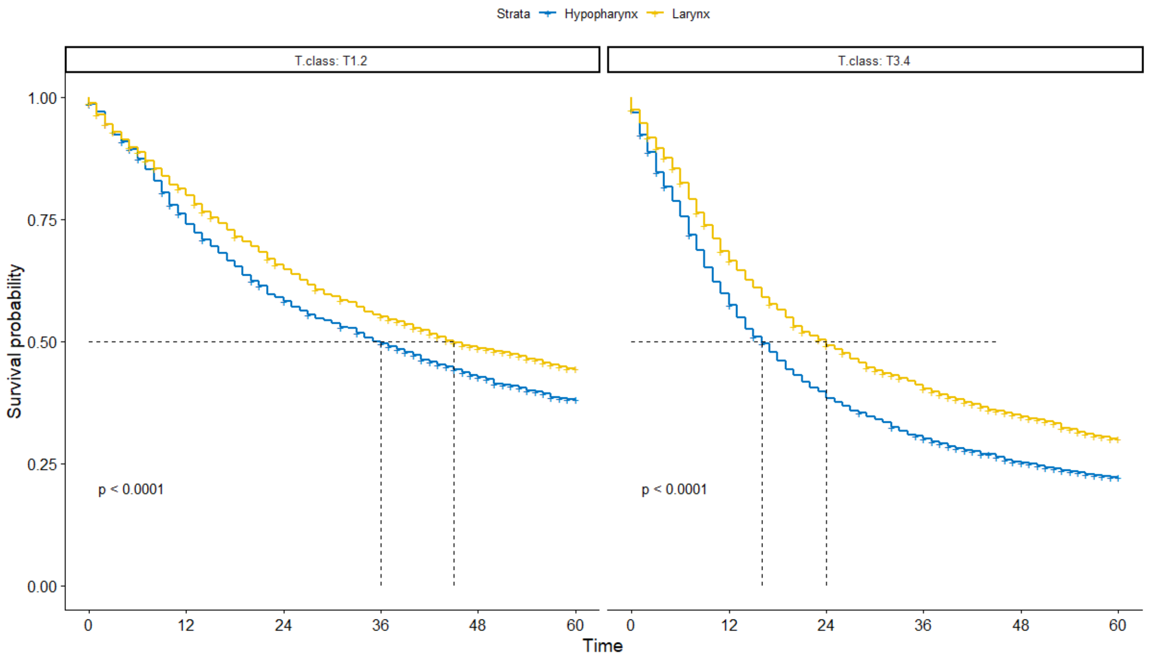

Supplement Figure 1. **The comparisons of overall survival time(OS) in 4256-pairs hypopharyngeal and laryngeal cancer patients**. After adjusting effects of age, gender, T classification, N classification, and AJCC clinical-stage, Kaplan-Meier analysis of OS in 4256 pairs of hypopharyngeal and laryngeal cancer patients showed that hypopharyngeal cancer patients remained survival shorter time than laryngeal cancer patients in early and late tumor-stage (all $p<0.01$).

Table S1								
ID	Accession	Gene Sym	HPM/LM		ID	Accession	Gene Sym	HPM/LM
1	IPI:IPI000	Gene_Sym	19.2309		58	IPI:IPI004	Gene_Sym	0.0356
2	IPI:IPI002	Gene_Sym	6.1944		59	IPI:IPI004	Gene_Sym	14.3219
3	IPI:IPI000	Gene_Sym	27.2898		60	IPI:IPI002	Gene_Sym	2.3988
4	IPI:IPI004	Gene_Sym	4.9204		61	IPI:IPI000	Gene_Sym	20.3236
5	IPI:IPI003	Gene_Sym	2.3988		62	IPI:IPI000	Gene_Sym	20.1372
6	IPI:IPI000	Gene_Sym	4.2462		63	IPI:IPI003	Gene_Sym	13.4277
7	IPI:IPI003	Gene_Sym	6.9823		64	IPI:IPI000	Gene_Sym	14.8594
9	IPI:IPI000	Gene_Sym	4.7424		65	IPI:IPI004	Gene_Sym	20.3236
10	IPI:IPI000	Gene_Sym	13.4277		66	IPI:IPI003	Gene_Sym	7.8705
11	IPI:IPI002	Gene_Sym	16.1436		67	IPI:IPI008	Gene_Sym	8.7096
12	IPI:IPI002	Gene_Sym	3.1915		68	IPI:IPI002	Gene_Sym	18.8799
14	IPI:IPI000	Gene_Sym	14.5881		69	IPI:IPI006	Gene_Sym	1.3428
15	IPI:IPI007	Gene_Sym	1.2823		70	IPI:IPI000	Gene_Sym	19.5884
16	IPI:IPI008	Gene_Sym	0.7586		71	IPI:IPI000	Gene_Sym	11.8032
17	IPI:IPI004	Gene_Sym	4.9204		72	IPI:IPI003	Gene_Sym	12.2462
18	IPI:IPI000	Gene_Sym	8.091		73	IPI:IPI001	Gene_Sym	13.1826
19	IPI:IPI000	Gene_Sym	2.1878		74	IPI:IPI002	Gene_Sym	9.6383
20	IPI:IPI007	Gene_Sym	7.4473		75	IPI:IPI003	Gene_Sym	15.8489
21	IPI:IPI003	Gene_Sym	11.9124		76	IPI:IPI006	Gene_Sym	7.9433
23	IPI:IPI005	Gene_Sym	10.9648		77	IPI:IPI000	Gene_Sym	18.3654
24	IPI:IPI008	Gene_Sym	17.0608		78	IPI:IPI008	Gene_Sym	4.2073
25	IPI:IPI000	Gene_Sym	14.0605		79	IPI:IPI003	Gene_Sym	0.4093
26	IPI:IPI004	Gene_Sym	35.9749		80	IPI:IPI006	Gene_Sym	6.9823
27	IPI:IPI000	Gene_Sym	19.0546		81	IPI:IPI007	Gene_Sym	12.5892
28	IPI:IPI008	Gene_Sym	7.0469		82	IPI:IPI006	Gene_Sym	3.7325
29	IPI:IPI004	Gene_Sym	14.4544		83	IPI:IPI003	Gene_Sym	3.02
30	IPI:IPI000	Gene_Sym	6.9183		84	IPI:IPI000	Gene_Sym	4.9659
31	IPI:IPI002	Gene_Sym	3.0479		85	IPI:IPI005	Gene_Sym	5.445
32	IPI:IPI004	Gene_Sym	13.9316		86	IPI:IPI002	Gene_Sym	3.7325
33	IPI:IPI008	Gene_Sym	12.5892		87	IPI:IPI005	Gene_Sym	12.942
34	IPI:IPI004	Gene_Sym	6.4863		88	IPI:IPI000	Gene_Sym	2.7542
35	IPI:IPI000	Gene_Sym	5.445		89	IPI:IPI000	Gene_Sym	9.3756
36	IPI:IPI000	Gene_Sym	8.7096		90	IPI:IPI000	Gene_Sym	3.8371
37	IPI:IPI008	Gene_Sym	11.695		91	IPI:IPI007	Gene_Sym	8.2414
38	IPI:IPI002	Gene_Sym	0.6081		92	IPI:IPI000	Gene_Sym	14.0605
39	IPI:IPI002	Gene_Sym	5.1523		93	IPI:IPI002	Gene_Sym	10.3753
40	IPI:IPI004	Gene_Sym	12.8233		94	IPI:IPI002	Gene_Sym	1.6144
42	IPI:IPI000	Gene_Sym	13.1826		95	IPI:IPI001	Gene_Sym	11.272
43	IPI:IPI004	Gene_Sym	86.2979		96	IPI:IPI002	Gene_Sym	4.2073
44	IPI:IPI003	Gene_Sym	29.3765		97	IPI:IPI000	Gene_Sym	5.4954
45	IPI:IPI000	Gene_Sym	15.417		98	IPI:IPI002	Gene_Sym	5.3456
46	IPI:IPI003	Gene_Sym	17.8649		99	IPI:IPI008	Gene_Sym	9.9083
47	IPI:IPI008	Gene_Sym	6.7298		100	IPI:IPI007	Gene_Sym	10.7647
48	IPI:IPI000	Gene_Sym	0.4875		101	IPI:IPI008	Gene_Sym	21.2814
49	IPI:IPI000	Gene_Sym	5.2966		102	IPI:IPI008	Gene_Sym	40.9261
50	IPI:IPI005	Gene_Sym	52.4808		103	IPI:IPI000	Gene_Sym	5.6494
52	IPI:IPI002	Gene_Sym	11.272		105	IPI:IPI000	Gene_Sym	1.2134
54	IPI:IPI000	Gene_Sym	5.7016		106	IPI:IPI004	Gene_Sym	1.8197
55	IPI:IPI004	Gene_Sym	5.3951		107	IPI:IPI002	Gene_Sym	19.7697
56	IPI:IPI007	Gene_Sym	24.6604		108	IPI:IPI002	Gene_Sym	10.4713
57	IPI:IPI002	Gene_Sym	6.3096		109	IPI:IPI000	Gene_Sym	8.8716
111	IPI:IPI002	Gene_Sym	3.1333		110	IPI:IPI007	Gene_Sym	0.6081
112	IPI:IPI006	Gene_Sym	4.9659		167	IPI:IPI007	Gene_Sym	6.4269
113	IPI:IPI006	Gene_Sym	6.6681		168	IPI:IPI004	Gene_Sym	32.5087

115	IPI:IPI002	Gene_Sym	4.5709		169	IPI:IPI000	Gene_Sym	39.0841
116	IPI:IPI000	Gene_Sym	9.2897		170	IPI:IPI006	Gene_Sym	2.4889
117	IPI:IPI007	Gene_Sym	4.0551		172	IPI:IPI000	Gene_Sym	8.3176
118	IPI:IPI001	Gene_Sym	25.5859		173	IPI:IPI000	Gene_Sym	2.208
119	IPI:IPI004	Gene_Sym	15.417		174	IPI:IPI000	Gene_Sym	8.0168
120	IPI:IPI008	Gene_Sym	0.133		176	IPI:IPI004	Gene_Sym	14.0605
121	IPI:IPI001	Gene_Sym	6.2517		177	IPI:IPI003	Gene_Sym	3.3419
122	IPI:IPI007	Gene_Sym	1.7865		178	IPI:IPI007	Gene_Sym	9.5499
123	IPI:IPI002	Gene_Sym	35.3183		179	IPI:IPI000	Gene_Sym	87.0964
124	IPI:IPI008	Gene_Sym	10.0925		180	IPI:IPI008	Gene_Sym	30.1995
125	IPI:IPI000	Gene_Sym	8.2414		181	IPI:IPI004	Gene_Sym	1.3062
126	IPI:IPI002	Gene_Sym	6.0256		182	IPI:IPI000	Gene_Sym	32.8095
127	IPI:IPI002	Gene_Sym	22.4905		183	IPI:IPI000	Gene_Sym	81.6582
128	IPI:IPI000	Gene_Sym	10.2802		184	IPI:IPI000	Gene_Sym	30.479
129	IPI:IPI002	Gene_Sym	99.0832		185	IPI:IPI002	Gene_Sym	8.6298
130	IPI:IPI000	Gene_Sym	1.7865		186	IPI:IPI002	Gene_Sym	99.0832
131	IPI:IPI000	Gene_Sym	14.3219		187	IPI:IPI002	Gene_Sym	18.7068
132	IPI:IPI008	Gene_Sym	1.5704		188	IPI:IPI002	Gene_Sym	10.4713
133	IPI:IPI006	Gene_Sym	5.3951		189	IPI:IPI002	Gene_Sym	24.2103
134	IPI:IPI007	Gene_Sym	7.1779		190	IPI:IPI000	Gene_Sym	15.2757
135	IPI:IPI000	Gene_Sym	38.0189		191	IPI:IPI005	Gene_Sym	19.4089
136	IPI:IPI002	Gene_Sym	1.4322		192	IPI:IPI003	Gene_Sym	19.4089
137	IPI:IPI002	Gene_Sym	3.4041		193	IPI:IPI000	Gene_Sym	5.0119
138	IPI:IPI004	Gene_Sym	0.057		194	IPI:IPI002	Gene_Sym	22.9087
139	IPI:IPI008	Gene_Sym	16.9044		195	IPI:IPI000	Gene_Sym	2.355
140	IPI:IPI004	Gene_Sym	3.5975		196	IPI:IPI002	Gene_Sym	8.2414
141	IPI:IPI000	Gene_Sym	32.5087		197	IPI:IPI002	Gene_Sym	0.7311
143	IPI:IPI004	Gene_Sym	2.5351		198	IPI:IPI002	Gene_Sym	18.0302
144	IPI:IPI001	Gene_Sym	4.8306		199	IPI:IPI000	Gene_Sym	11.5878
145	IPI:IPI002	Gene_Sym	4.7863		201	IPI:IPI000	Gene_Sym	13.9316
146	IPI:IPI008	Gene_Sym	17.5388		202	IPI:IPI003	Gene_Sym	10.666
147	IPI:IPI000	Gene_Sym	3.767		203	IPI:IPI000	Gene_Sym	30.1995
149	IPI:IPI007	Gene_Sym	13.4277		204	IPI:IPI000	Gene_Sym	8.3176
150	IPI:IPI000	Gene_Sym	2.884		205	IPI:IPI000	Gene_Sym	23.5505
151	IPI:IPI007	Gene_Sym	12.4738		206	IPI:IPI002	Gene_Sym	0.1294
152	IPI:IPI004	Gene_Sym	3.1623		207	IPI:IPI000	Gene_Sym	1.3677
153	IPI:IPI000	Gene_Sym	21.677		209	IPI:IPI004	Gene_Sym	3.2211
154	IPI:IPI001	Gene_Sym	8.0168		211	IPI:IPI007	Gene_Sym	1.803
155	IPI:IPI004	Gene_Sym	23.7684		212	IPI:IPI007	Gene_Sym	0.2559
156	IPI:IPI000	Gene_Sym	0.4406		213	IPI:IPI004	Gene_Sym	0.6668
157	IPI:IPI008	Gene_Sym	47.4242		215	IPI:IPI002	Gene_Sym	3.9446
158	IPI:IPI002	Gene_Sym	16.7494		216	IPI:IPI002	Gene_Sym	0.5058
159	IPI:IPI002	Gene_Sym	8.3176		217	IPI:IPI002	Gene_Sym	0.2489
160	IPI:IPI000	Gene_Sym	4.9659		218	IPI:IPI008	Gene_Sym	87.9023
162	IPI:IPI000	Gene_Sym	17.5388		219	IPI:IPI000	Gene_Sym	7.1121
163	IPI:IPI002	Gene_Sym	31.3329		220	IPI:IPI002	Gene_Sym	0.2109
164	IPI:IPI005	Gene_Sym	2.208		221	IPI:IPI002	Gene_Sym	18.197
165	IPI:IPI000	Gene_Sym	42.0727		222	IPI:IPI000	Gene_Sym	38.7258
166	IPI:IPI008	Gene_Sym	19.4089		223	IPI:IPI000	Gene_Sym	99.0832
228	IPI:IPI008	Gene_Sym	13.3045		224	IPI:IPI000	Gene_Sym	16.5959
229	IPI:IPI003	Gene_Sym	21.677		294	IPI:IPI000	Gene_Sym	12.4738
230	IPI:IPI007	Gene_Sym	9.2045		295	IPI:IPI004	Gene_Sym	0.0575
231	IPI:IPI003	Gene_Sym	16.5959		296	IPI:IPI001	Gene_Sym	8.8716
232	IPI:IPI000	Gene_Sym	9.2045		297	IPI:IPI008	Gene_Sym	87.0964
234	IPI:IPI000	Gene_Sym	3.767		299	IPI:IPI000	Gene_Sym	2.9107
235	IPI:IPI000	Gene_Sym	12.0226		300	IPI:IPI002	Gene_Sym	99.0832

237	IPI:IPI004	Gene_Sym	87.9023		301	IPI:IPI000	Gene_Sym	18.8799
238	IPI:IPI007	Gene_Sym	6.2517		302	IPI:IPI002	Gene_Sym	99.0832
240	IPI:IPI002	Gene_Sym	7.9433		303	IPI:IPI000	Gene_Sym	2.208
241	IPI:IPI008	Gene_Sym	19.7697		304	IPI:IPI007	Gene_Sym	99.0832
242	IPI:IPI000	Gene_Sym	1.2589		305	IPI:IPI000	Gene_Sym	87.9023
243	IPI:IPI001	Gene_Sym	2.9376		306	IPI:IPI007	Gene_Sym	87.9023
244	IPI:IPI004	Gene_Sym	38.0189		307	IPI:IPI004	Gene_Sym	16.1436
247	IPI:IPI002	Gene_Sym	10.666		308	IPI:IPI005	Gene_Sym	29.9226
248	IPI:IPI006	Gene_Sym	87.9023		309	IPI:IPI007	Gene_Sym	11.695
249	IPI:IPI000	Gene_Sym	4.7863		310	IPI:IPI002	Gene_Sym	6.5464
250	IPI:IPI000	Gene_Sym	1.9409		311	IPI:IPI000	Gene_Sym	0.6252
251	IPI:IPI008	Gene_Sym	87.9023		312	IPI:IPI008	Gene_Sym	87.9023
252	IPI:IPI007	Gene_Sym	17.378		313	IPI:IPI000	Gene_Sym	18.8799
253	IPI:IPI000	Gene_Sym	0.0887		316	IPI:IPI006	Gene_Sym	29.9226
254	IPI:IPI002	Gene_Sym	1.6749		318	IPI:IPI002	Gene_Sym	8.1658
255	IPI:IPI002	Gene_Sym	0.7586		319	IPI:IPI000	Gene_Sym	13.4277
256	IPI:IPI002	Gene_Sym	0.7178		320	IPI:IPI000	Gene_Sym	0.0244
257	IPI:IPI000	Gene_Sym	4.6559		321	IPI:IPI000	Gene_Sym	19.7697
258	IPI:IPI000	Gene_Sym	87.9023		322	IPI:IPI005	Gene_Sym	44.0555
259	IPI:IPI002	Gene_Sym	0.2377		323	IPI:IPI008	Gene_Sym	87.9023
260	IPI:IPI004	Gene_Sym	87.9023		324	IPI:IPI000	Gene_Sym	0.3499
261	IPI:IPI000	Gene_Sym	7.5162		325	IPI:IPI007	Gene_Sym	22.9087
262	IPI:IPI000	Gene_Sym	26.7917		326	IPI:IPI008	Gene_Sym	18.0302
263	IPI:IPI000	Gene_Sym	99.0832		327	IPI:IPI005	Gene_Sym	8.1658
265	IPI:IPI002	Gene_Sym	10.666		330	IPI:IPI005	Gene_Sym	34.0408
267	IPI:IPI000	Gene_Sym	3.5318		331	IPI:IPI008	Gene_Sym	5.1523
269	IPI:IPI007	Gene_Sym	0.6792		333	IPI:IPI000	Gene_Sym	99.0832
270	IPI:IPI008	Gene_Sym	0.2188		334	IPI:IPI000	Gene_Sym	1.4454
271	IPI:IPI000	Gene_Sym	99.0832		335	IPI:IPI004	Gene_Sym	0.2606
273	IPI:IPI002	Gene_Sym	0.0766		336	IPI:IPI000	Gene_Sym	6.9823
274	IPI:IPI008	Gene_Sym	87.9023		337	IPI:IPI004	Gene_Sym	17.8649
275	IPI:IPI002	Gene_Sym	87.9023		338	IPI:IPI004	Gene_Sym	7.2444
276	IPI:IPI000	Gene_Sym	1.803		340	IPI:IPI007	Gene_Sym	87.9023
277	IPI:IPI000	Gene_Sym	1.7701		342	IPI:IPI008	Gene_Sym	2.3121
278	IPI:IPI000	Gene_Sym	7.656		343	IPI:IPI003	Gene_Sym	87.9023
279	IPI:IPI006	Gene_Sym	24.6604		344	IPI:IPI002	Gene_Sym	31.3329
281	IPI:IPI000	Gene_Sym	2.8314		346	IPI:IPI003	Gene_Sym	19.9526
282	IPI:IPI006	Gene_Sym	20.1372		348	IPI:IPI000	Gene_Sym	7.1121
285	IPI:IPI000	Gene_Sym	2.1281		350	IPI:IPI000	Gene_Sym	87.9023
286	IPI:IPI000	Gene_Sym	0.6982		351	IPI:IPI000	Gene_Sym	17.2187
288	IPI:IPI008	Gene_Sym	75.8578		352	IPI:IPI000	Gene_Sym	1.4191
289	IPI:IPI000	Gene_Sym	26.3027		353	IPI:IPI000	Gene_Sym	28.3139
290	IPI:IPI004	Gene_Sym	1.6293		354	IPI:IPI007	Gene_Sym	12.0226
292	IPI:IPI008	Gene_Sym	1.5704		355	IPI:IPI008	Gene_Sym	1.9588
293	IPI:IPI008	Gene_Sym	8.2414		356	IPI:IPI000	Gene_Sym	0.5598
359	IPI:IPI000	Gene_Sym	4.4463		358	IPI:IPI008	Gene_Sym	0.2911
360	IPI:IPI005	Gene_Sym	87.9023		421	IPI:IPI007	Gene_Sym	4.6132
361	IPI:IPI000	Gene_Sym	0.6668		423	IPI:IPI000	Gene_Sym	0.1738
362	IPI:IPI002	Gene_Sym	0.3698		424	IPI:IPI002	Gene_Sym	3.0479
363	IPI:IPI002	Gene_Sym	34.9945		425	IPI:IPI000	Gene_Sym	99.0832
364	IPI:IPI000	Gene_Sym	11.3763		428	IPI:IPI002	Gene_Sym	2.2491
365	IPI:IPI000	Gene_Sym	5.5463		431	IPI:IPI004	Gene_Sym	8.6298
367	IPI:IPI007	Gene_Sym	99.0832		432	IPI:IPI000	Gene_Sym	0.2421
370	IPI:IPI003	Gene_Sym	4.2462		433	IPI:IPI007	Gene_Sym	99.0832
371	IPI:IPI001	Gene_Sym	86.2979		434	IPI:IPI004	Gene_Sym	0.4093
373	IPI:IPI002	Gene_Sym	10.666		435	IPI:IPI000	Gene_Sym	1.2942

374	IPI:IPI004	Gene_Sym	87.0964		436	IPI:IPI000	Gene_Sym	87.9023
375	IPI:IPI008	Gene_Sym	1.406		437	IPI:IPI008	Gene_Sym	0.3698
376	IPI:IPI008	Gene_Sym	10.2802		439	IPI:IPI001	Gene_Sym	27.2898
378	IPI:IPI004	Gene_Sym	6.3096		441	IPI:IPI005	Gene_Sym	2.9376
379	IPI:IPI008	Gene_Sym	1.4997		442	IPI:IPI000	Gene_Sym	0.6607
380	IPI:IPI002	Gene_Sym	14.1906		444	IPI:IPI000	Gene_Sym	1.8197
381	IPI:IPI001	Gene_Sym	24.4343		445	IPI:IPI003	Gene_Sym	22.6987
382	IPI:IPI003	Gene_Sym	44.4631		447	IPI:IPI008	Gene_Sym	0.0565
383	IPI:IPI003	Gene_Sym	11.272		448	IPI:IPI006	Gene_Sym	2.7542
384	IPI:IPI000	Gene_Sym	87.9023		449	IPI:IPI000	Gene_Sym	1.2589
385	IPI:IPI000	Gene_Sym	13.3045		450	IPI:IPI002	Gene_Sym	0.4571
386	IPI:IPI004	Gene_Sym	99.0832		451	IPI:IPI007	Gene_Sym	86.2979
387	IPI:IPI003	Gene_Sym	1.9953		452	IPI:IPI008	Gene_Sym	18.0302
388	IPI:IPI000	Gene_Sym	4.8753		453	IPI:IPI000	Gene_Sym	1.2023
389	IPI:IPI000	Gene_Sym	0.3162		454	IPI:IPI000	Gene_Sym	1.2706
390	IPI:IPI007	Gene_Sym	2.1677		455	IPI:IPI002	Gene_Sym	1.6749
391	IPI:IPI004	Gene_Sym	4.8753		456	IPI:IPI003	Gene_Sym	9.8175
392	IPI:IPI002	Gene_Sym	87.9023		457	IPI:IPI002	Gene_Sym	13.6773
393	IPI:IPI000	Gene_Sym	0.3733		458	IPI:IPI000	Gene_Sym	87.9023
394	IPI:IPI006	Gene_Sym	0.6668		460	IPI:IPI000	Gene_Sym	87.9023
395	IPI:IPI003	Gene_Sym	4.3652		461	IPI:IPI000	Gene_Sym	29.1072
396	IPI:IPI007	Gene_Sym	34.6737		462	IPI:IPI008	Gene_Sym	0.6486
397	IPI:IPI007	Gene_Sym	1.3932		464	IPI:IPI002	Gene_Sym	2.5351
400	IPI:IPI007	Gene_Sym	6.8549		466	IPI:IPI002	Gene_Sym	87.9023
401	IPI:IPI004	Gene_Sym	6.6681		471	IPI:IPI000	Gene_Sym	15.2757
402	IPI:IPI002	Gene_Sym	4.5709		472	IPI:IPI001	Gene_Sym	1.3305
403	IPI:IPI001	Gene_Sym	7.7268		473	IPI:IPI005	Gene_Sym	7.1121
405	IPI:IPI002	Gene_Sym	5.2966		474	IPI:IPI000	Gene_Sym	87.9023
406	IPI:IPI000	Gene_Sym	99.0832		475	IPI:IPI002	Gene_Sym	3.3113
407	IPI:IPI002	Gene_Sym	0.7311		476	IPI:IPI000	Gene_Sym	1.5417
408	IPI:IPI000	Gene_Sym	0.0982		479	IPI:IPI003	Gene_Sym	10.2802
409	IPI:IPI007	Gene_Sym	87.9023		480	IPI:IPI007	Gene_Sym	48.3059
410	IPI:IPI000	Gene_Sym	0.0824		482	IPI:IPI000	Gene_Sym	5.2
411	IPI:IPI007	Gene_Sym	2.3768		483	IPI:IPI000	Gene_Sym	14.4544
412	IPI:IPI006	Gene_Sym	0.0711		484	IPI:IPI007	Gene_Sym	10.1859
413	IPI:IPI000	Gene_Sym	1.9409		485	IPI:IPI000	Gene_Sym	16.7494
414	IPI:IPI000	Gene_Sym	2.6062		486	IPI:IPI002	Gene_Sym	1.6144
415	IPI:IPI001	Gene_Sym	6.9823		488	IPI:IPI000	Gene_Sym	87.9023
416	IPI:IPI000	Gene_Sym	69.1831		489	IPI:IPI004	Gene_Sym	0.3467
418	IPI:IPI002	Gene_Sym	87.9023		490	IPI:IPI004	Gene_Sym	58.0764
419	IPI:IPI004	Gene_Sym	0.492		494	IPI:IPI000	Gene_Sym	3.9084
498	IPI:IPI008	Gene_Sym	0.6081		497	IPI:IPI005	Gene_Sym	24.4343
499	IPI:IPI000	Gene_Sym	99.0832		577	IPI:IPI002	Gene_Sym	5.3456
502	IPI:IPI003	Gene_Sym	0.5546		580	IPI:IPI000	Gene_Sym	87.9023
506	IPI:IPI008	Gene_Sym	0.0705		582	IPI:IPI003	Gene_Sym	0.5248
508	IPI:IPI001	Gene_Sym	0.6486		585	IPI:IPI000	Gene_Sym	10.666
510	IPI:IPI002	Gene_Sym	87.9023		586	IPI:IPI000	Gene_Sym	87.9023
511	IPI:IPI000	Gene_Sym	1.3932		588	IPI:IPI007	Gene_Sym	87.9023
512	IPI:IPI008	Gene_Sym	4.8753		589	IPI:IPI000	Gene_Sym	1.3062
513	IPI:IPI004	Gene_Sym	4.1305		590	IPI:IPI000	Gene_Sym	87.9023
515	IPI:IPI000	Gene_Sym	12.942		591	IPI:IPI008	Gene_Sym	0.5808
518	IPI:IPI008	Gene_Sym	87.9023		592	IPI:IPI002	Gene_Sym	0.3467
520	IPI:IPI003	Gene_Sym	87.9023		593	IPI:IPI002	Gene_Sym	87.9023
521	IPI:IPI000	Gene_Sym	87.9023		596	IPI:IPI000	Gene_Sym	0.302
522	IPI:IPI000	Gene_Sym	99.0832		599	IPI:IPI000	Gene_Sym	28.8403
523	IPI:IPI002	Gene_Sym	2.3768		600	IPI:IPI002	Gene_Sym	0.263

524	IPI:IPI007	Gene_Sym	20.3236		604	IPI:IPI003	Gene_Sym	0.5395
527	IPI:IPI006	Gene_Sym	42.8549		605	IPI:IPI008	Gene_Sym	0.7586
529	IPI:IPI003	Gene_Sym	2.355		607	IPI:IPI004	Gene_Sym	87.9023
530	IPI:IPI000	Gene_Sym	0.5861		608	IPI:IPI000	Gene_Sym	99.0832
531	IPI:IPI007	Gene_Sym	99.0832		609	IPI:IPI007	Gene_Sym	22.6987
532	IPI:IPI002	Gene_Sym	15.8489		612	IPI:IPI002	Gene_Sym	10.9648
533	IPI:IPI000	Gene_Sym	6.9183		613	IPI:IPI000	Gene_Sym	78.7046
535	IPI:IPI008	Gene_Sym	9.2045		616	IPI:IPI008	Gene_Sym	27.0396
536	IPI:IPI002	Gene_Sym	1.5136		620	IPI:IPI002	Gene_Sym	0.055
540	IPI:IPI000	Gene_Sym	1.8535		631	IPI:IPI001	Gene_Sym	4.6989
541	IPI:IPI000	Gene_Sym	6.9823		632	IPI:IPI004	Gene_Sym	14.7231
542	IPI:IPI000	Gene_Sym	0.1644		633	IPI:IPI002	Gene_Sym	87.9023
543	IPI:IPI007	Gene_Sym	77.983		634	IPI:IPI000	Gene_Sym	6.368
544	IPI:IPI004	Gene_Sym	73.1139		637	IPI:IPI000	Gene_Sym	87.9023
545	IPI:IPI008	Gene_Sym	60.8135		638	IPI:IPI003	Gene_Sym	8.6298
547	IPI:IPI003	Gene_Sym	87.9023		640	IPI:IPI000	Gene_Sym	14.8594
548	IPI:IPI000	Gene_Sym	19.7697		642	IPI:IPI008	Gene_Sym	30.761
549	IPI:IPI007	Gene_Sym	1.4191		644	IPI:IPI007	Gene_Sym	4.6132
551	IPI:IPI007	Gene_Sym	99.0832		647	IPI:IPI008	Gene_Sym	1.9953
553	IPI:IPI006	Gene_Sym	0.6855		648	IPI:IPI000	Gene_Sym	0.6792
554	IPI:IPI007	Gene_Sym	0.6855		649	IPI:IPI008	Gene_Sym	87.9023
555	IPI:IPI000	Gene_Sym	0.0377		650	IPI:IPI008	Gene_Sym	0.5248
556	IPI:IPI000	Gene_Sym	0.7798		654	IPI:IPI002	Gene_Sym	3.0479
557	IPI:IPI004	Gene_Sym	42.0727		655	IPI:IPI000	Gene_Sym	0.6194
558	IPI:IPI002	Gene_Sym	87.9023		656	IPI:IPI005	Gene_Sym	15.417
559	IPI:IPI007	Gene_Sym	4.529		662	IPI:IPI000	Gene_Sym	5.5463
560	IPI:IPI000	Gene_Sym	11.9124		665	IPI:IPI002	Gene_Sym	87.0964
561	IPI:IPI007	Gene_Sym	1.7061		667	IPI:IPI000	Gene_Sym	87.9023
562	IPI:IPI002	Gene_Sym	87.9023		671	IPI:IPI002	Gene_Sym	3.8019
563	IPI:IPI000	Gene_Sym	0.7311		673	IPI:IPI000	Gene_Sym	87.9023
566	IPI:IPI000	Gene_Sym	34.9945		674	IPI:IPI006	Gene_Sym	0.5702
569	IPI:IPI000	Gene_Sym	17.378		676	IPI:IPI002	Gene_Sym	74.4732
570	IPI:IPI000	Gene_Sym	1.7219		679	IPI:IPI000	Gene_Sym	0.1393
571	IPI:IPI000	Gene_Sym	20.3236		680	IPI:IPI000	Gene_Sym	87.9023
572	IPI:IPI002	Gene_Sym	10.1859		681	IPI:IPI004	Gene_Sym	0.1542
573	IPI:IPI008	Gene_Sym	0.4875		682	IPI:IPI008	Gene_Sym	0.7798
574	IPI:IPI000	Gene_Sym	13.8038		683	IPI:IPI000	Gene_Sym	1.5417
685	IPI:IPI002	Gene_Sym	3.3113		684	IPI:IPI002	Gene_Sym	3.3729
688	IPI:IPI000	Gene_Sym	4.1687		772	IPI:IPI000	Gene_Sym	0.6081
689	IPI:IPI000	Gene_Sym	0.3802		774	IPI:IPI008	Gene_Sym	39.8107
690	IPI:IPI006	Gene_Sym	0.2109		776	IPI:IPI000	Gene_Sym	87.9023
691	IPI:IPI000	Gene_Sym	0.5808		778	IPI:IPI004	Gene_Sym	87.9023
692	IPI:IPI000	Gene_Sym	87.9023		779	IPI:IPI002	Gene_Sym	87.9023
695	IPI:IPI003	Gene_Sym	12.942		781	IPI:IPI000	Gene_Sym	87.9023
697	IPI:IPI008	Gene_Sym	87.9023		785	IPI:IPI000	Gene_Sym	1.6904
699	IPI:IPI000	Gene_Sym	87.9023		786	IPI:IPI006	Gene_Sym	2.1086
700	IPI:IPI003	Gene_Sym	0.0698		787	IPI:IPI007	Gene_Sym	1.9953
703	IPI:IPI003	Gene_Sym	99.0832		788	IPI:IPI007	Gene_Sym	99.0832
704	IPI:IPI000	Gene_Sym	2.208		790	IPI:IPI008	Gene_Sym	1.5136
705	IPI:IPI000	Gene_Sym	0.6918		791	IPI:IPI000	Gene_Sym	59.7035
711	IPI:IPI005	Gene_Sym	87.9023		793	IPI:IPI007	Gene_Sym	86.2979
712	IPI:IPI000	Gene_Sym	87.9023		794	IPI:IPI006	Gene_Sym	0.6427
716	IPI:IPI000	Gene_Sym	1.8707		797	IPI:IPI000	Gene_Sym	0.6855
718	IPI:IPI004	Gene_Sym	52.9663		799	IPI:IPI000	Gene_Sym	75.8578
720	IPI:IPI000	Gene_Sym	0.2014		801	IPI:IPI000	Gene_Sym	1.406
721	IPI:IPI000	Gene_Sym	99.0832		802	IPI:IPI000	Gene_Sym	24.8886

723	IPI:IPI003	Gene_Sym	87.0964		805	IPI:IPI000	Gene_Sym	0.4966	
724	IPI:IPI002	Gene_Sym	2.0893		807	IPI:IPI004	Gene_Sym	17.5388	
726	IPI:IPI000	Gene_Sym	2.208		808	IPI:IPI000	Gene_Sym	2.3335	
728	IPI:IPI000	Gene_Sym	25.3513		810	IPI:IPI007	Gene_Sym	14.0605	
729	IPI:IPI002	Gene_Sym	87.9023		812	IPI:IPI000	Gene_Sym	18.5353	
730	IPI:IPI006	Gene_Sym	99.0832		814	IPI:IPI002	Gene_Sym	87.9023	
731	IPI:IPI006	Gene_Sym	86.2979		815	IPI:IPI000	Gene_Sym	0.3631	
734	IPI:IPI006	Gene_Sym	87.9023		817	IPI:IPI000	Gene_Sym	35.3183	
735	IPI:IPI006	Gene_Sym	6.792		821	IPI:IPI007	Gene_Sym	8.9536	
736	IPI:IPI004	Gene_Sym	35.9749		823	IPI:IPI000	Gene_Sym	1.2942	
737	IPI:IPI002	Gene_Sym	12.5892		826	IPI:IPI000	Gene_Sym	87.9023	
739	IPI:IPI000	Gene_Sym	0.0291		827	IPI:IPI006	Gene_Sym	0.6918	
740	IPI:IPI000	Gene_Sym	2.355		829	IPI:IPI006	Gene_Sym	1.7378	
742	IPI:IPI000	Gene_Sym	1.6293		830	IPI:IPI007	Gene_Sym	4.6132	
744	IPI:IPI000	Gene_Sym	0.4246		832	IPI:IPI007	Gene_Sym	99.0832	
746	IPI:IPI006	Gene_Sym	87.9023		833	IPI:IPI000	Gene_Sym	87.9023	
747	IPI:IPI002	Gene_Sym	87.9023		836	IPI:IPI000	Gene_Sym	0.5248	
748	IPI:IPI000	Gene_Sym	18.8799		838	IPI:IPI008	Gene_Sym	6.0256	
749	IPI:IPI003	Gene_Sym	0.037		841	IPI:IPI008	Gene_Sym	84.7227	
750	IPI:IPI000	Gene_Sym	87.9023		842	IPI:IPI000	Gene_Sym	0.4093	
752	IPI:IPI000	Gene_Sym	5.8076		844	IPI:IPI002	Gene_Sym	6.7298	
753	IPI:IPI000	Gene_Sym	13.6773		845	IPI:IPI001	Gene_Sym	4.7424	
754	IPI:IPI006	Gene_Sym	87.9023		848	IPI:IPI008	Gene_Sym	87.9023	
755	IPI:IPI000	Gene_Sym	0.4699		852	IPI:IPI000	Gene_Sym	87.9023	
756	IPI:IPI000	Gene_Sym	2.0512						
758	IPI:IPI000	Gene_Sym	1.406						
760	IPI:IPI008	Gene_Sym	0.6486						
761	IPI:IPI000	Gene_Sym	32.5087						
762	IPI:IPI002	Gene_Sym	87.9023						
764	IPI:IPI003	Gene_Sym	1.6596						
765	IPI:IPI008	Gene_Sym	0.1585						
769	IPI:IPI001	Gene_Sym	0.4406						
771	IPI:IPI003	Gene_Sym	87.9023						

Table S2

ID	Accession	Gene Symbo	HPM/LM
8	IPI:IPI00041	Gene_Symb	1.1695
22	IPI:IPI00030	Gene_Symb	1.1803
41	IPI:IPI00009	Gene_Symb	0.871
51	IPI:IPI00010	Gene_Symb	1.0186
53	IPI:IPI00017	Gene_Symb	1.1272
114	IPI:IPI00017	Gene_Symb	0.879
148	IPI:IPI00027	Gene_Symb	1.1912
161	IPI:IPI00008	Gene_Symb	1.1272
200	IPI:IPI00749	Gene_Symb	1.1169
208	IPI:IPI00005	Gene_Symb	1.0666
214	IPI:IPI00884	Gene_Symb	1.1695
283	IPI:IPI00304	Gene_Symb	1
284	IPI:IPI00642	Gene_Symb	0.912
287	IPI:IPI00002	Gene_Symb	1.1695
345	IPI:IPI00033	Gene_Symb	1.0666
368	IPI:IPI00295	Gene_Symb	0.9376
493	IPI:IPI00007	Gene_Symb	1.1588
514	IPI:IPI00027	Gene_Symb	0.8017
519	IPI:IPI00217	Gene_Symb	0.8551
528	IPI:IPI00000	Gene_Symb	1.1376
617	IPI:IPI00012	Gene_Symb	0.9638
639	IPI:IPI00794	Gene_Symb	1.0568
713	IPI:IPI00010	Gene_Symb	0.8551
714	IPI:IPI00550	Gene_Symb	0.9638
751	IPI:IPI00018	Gene_Symb	0.9036
759	IPI:IPI00298	Gene_Symb	0.8551
796	IPI:IPI00184	Gene_Symb	1
818	IPI:IPI00294	Gene_Symb	0.8872
828	IPI:IPI0087	Gene_Symb	1.0568
839	IPI:IPI00028	Gene_Symb	0.8954

Table S3

ID	Accession	Gene Symbo	HPM/LM	HPC/LC		ID	Accession	Gene Symbo	HPM/LM	HPC/LC
4	IPI:IPI0042	Gene_Symb	4.9204	0.5754		165	IPI:IPI0001	Gene_Symb	42.0727	0.5395
6	IPI:IPI0000	Gene_Symb	4.2462	0.597		166	IPI:IPI0082	Gene_Symb	19.4089	0.6668
8	IPI:IPI0041	Gene_Symb	1.1695	1.2706		168	IPI:IPI0047	Gene_Symb	32.5087	0.7586
9	IPI:IPI0001	Gene_Symb	4.7424	0.302		170	IPI:IPI0064	Gene_Symb	2.4889	0.4406
12	IPI:IPI0021	Gene_Symb	3.1915	0.078		173	IPI:IPI0002	Gene_Symb	2.208	0.597
17	IPI:IPI0045	Gene_Symb	4.9204	0.0759		182	IPI:IPI0000	Gene_Symb	32.8095	0.7244
18	IPI:IPI0002	Gene_Symb	8.091	0.3076		184	IPI:IPI0003	Gene_Symb	30.479	0.6918
20	IPI:IPI0078	Gene_Symb	7.4473	0.5105		187	IPI:IPI0021	Gene_Symb	18.7068	0.7047
27	IPI:IPI0002	Gene_Symb	19.0546	0.7244		193	IPI:IPI0003	Gene_Symb	5.0119	0.787
28	IPI:IPI0085	Gene_Symb	7.0469	0.4699		194	IPI:IPI0022	Gene_Symb	22.9087	0.7798
30	IPI:IPI0001	Gene_Symb	6.9183	0.6252		197	IPI:IPI0029	Gene_Symb	0.7311	1.2359
33	IPI:IPI0086	Gene_Symb	12.5892	0.6026		205	IPI:IPI0001	Gene_Symb	23.5505	0.2512
34	IPI:IPI0047	Gene_Symb	6.4863	0.4246		206	IPI:IPI0029	Gene_Symb	0.1294	31.0456
41	IPI:IPI0000	Gene_Symb	0.871	0.1225		211	IPI:IPI0079	Gene_Symb	1.803	0.6792
43	IPI:IPI0043	Gene_Symb	86.2979	0.5495		213	IPI:IPI0047	Gene_Symb	0.6668	2.5351
49	IPI:IPI0001	Gene_Symb	5.2966	0.4055		214	IPI:IPI0088	Gene_Symb	1.1695	1.4723
51	IPI:IPI0001	Gene_Symb	1.0186	0.177		216	IPI:IPI0021	Gene_Symb	0.5058	6.7298
52	IPI:IPI0021	Gene_Symb	11.272	0.6607		220	IPI:IPI0021	Gene_Symb	0.2109	13.4277
54	IPI:IPI0007	Gene_Symb	5.7016	0.1803		221	IPI:IPI0022	Gene_Symb	18.197	0.6918
58	IPI:IPI0046	Gene_Symb	0.0356	19.7697		222	IPI:IPI0001	Gene_Symb	38.7258	0.7516
60	IPI:IPI0021	Gene_Symb	2.3988	0.6026		224	IPI:IPI0003	Gene_Symb	16.5959	0.4446
61	IPI:IPI0000	Gene_Symb	20.3236	0.6982		232	IPI:IPI0002	Gene_Symb	9.2045	0.6668
62	IPI:IPI0002	Gene_Symb	20.1372	0.7727		234	IPI:IPI0000	Gene_Symb	3.767	0.7656
63	IPI:IPI0039	Gene_Symb	13.4277	0.7112		237	IPI:IPI0045	Gene_Symb	87.9023	0.7656
66	IPI:IPI0038	Gene_Symb	7.8705	0.6368		238	IPI:IPI0078	Gene_Symb	6.2517	0.6792
67	IPI:IPI0084	Gene_Symb	8.7096	0.1556		240	IPI:IPI0029	Gene_Symb	7.9433	0.4742
73	IPI:IPI0018	Gene_Symb	13.1826	0.4207		243	IPI:IPI0016	Gene_Symb	2.9376	0.2754
74	IPI:IPI0022	Gene_Symb	9.6383	0.5861		249	IPI:IPI0001	Gene_Symb	4.7863	0.4018
76	IPI:IPI0064	Gene_Symb	7.9433	0.4656		250	IPI:IPI0002	Gene_Symb	1.9409	0.4446
77	IPI:IPI0000	Gene_Symb	18.3654	0.7178		254	IPI:IPI0029	Gene_Symb	1.6749	0.2148
80	IPI:IPI0060	Gene_Symb	6.9823	0.5916		255	IPI:IPI0029	Gene_Symb	0.7586	1.7378
82	IPI:IPI0064	Gene_Symb	3.7325	0.673		263	IPI:IPI0003	Gene_Symb	99.0832	0.6194
84	IPI:IPI0000	Gene_Symb	4.9659	0.3733		270	IPI:IPI0088	Gene_Symb	0.2188	9.2897
85	IPI:IPI0055	Gene_Symb	5.445	0.133		273	IPI:IPI0021	Gene_Symb	0.0766	37.325
86	IPI:IPI0021	Gene_Symb	3.7325	0.7943		276	IPI:IPI0002	Gene_Symb	1.803	0.6792
87	IPI:IPI0055	Gene_Symb	12.942	0.2938		277	IPI:IPI0002	Gene_Symb	1.7701	0.5395
91	IPI:IPI0078	Gene_Symb	8.2414	0.4966		278	IPI:IPI0001	Gene_Symb	7.656	0.3105
93	IPI:IPI0029	Gene_Symb	10.3753	0.2606		279	IPI:IPI0064	Gene_Symb	24.6604	0.787
94	IPI:IPI0023	Gene_Symb	1.6144	0.5861		281	IPI:IPI0000	Gene_Symb	2.8314	0.7586
97	IPI:IPI0001	Gene_Symb	5.4954	0.7047		283	IPI:IPI0030	Gene_Symb	1	1.3305
98	IPI:IPI0029	Gene_Symb	5.3456	0.5012		284	IPI:IPI0064	Gene_Symb	0.912	0.7047
109	IPI:IPI0002	Gene_Symb	8.8716	0.6792		287	IPI:IPI0000	Gene_Symb	1.1695	6.1376
110	IPI:IPI0073	Gene_Symb	0.6081	1.6904		288	IPI:IPI0083	Gene_Symb	75.8578	0.673
112	IPI:IPI0064	Gene_Symb	4.9659	0.7379		292	IPI:IPI0087	Gene_Symb	1.5704	0.787
114	IPI:IPI0017	Gene_Symb	0.879	4.2462		295	IPI:IPI0047	Gene_Symb	0.0575	12.7057
115	IPI:IPI0029	Gene_Symb	4.5709	0.7311		297	IPI:IPI0087	Gene_Symb	87.0964	0.7943
116	IPI:IPI0001	Gene_Symb	9.2897	0.4571		301	IPI:IPI0002	Gene_Symb	18.8799	0.5445
120	IPI:IPI0087	Gene_Symb	0.133	7.8705		303	IPI:IPI0002	Gene_Symb	2.208	0.3597

121	IPI:IPI0018	Gene_Symb	6.2517	0.6855		308	IPI:IPI0051	Gene_Symb	29.9226	0.7943
124	IPI:IPI0087	Gene_Symb	10.0925	0.4365		309	IPI:IPI0074	Gene_Symb	11.695	0.7047
128	IPI:IPI0001	Gene_Symb	10.2802	0.6607		310	IPI:IPI0021	Gene_Symb	6.5464	0.5058
138	IPI:IPI0041	Gene_Symb	0.057	2.5119		312	IPI:IPI0088	Gene_Symb	87.9023	0.6982
140	IPI:IPI0040	Gene_Symb	3.5975	0.4093		318	IPI:IPI0029	Gene_Symb	8.1658	0.673
143	IPI:IPI0041	Gene_Symb	2.5351	0.6252		321	IPI:IPI0001	Gene_Symb	19.7697	0.7516
148	IPI:IPI0002	Gene_Symb	1.1912	1.4322		324	IPI:IPI0000	Gene_Symb	0.3499	1.4997
151	IPI:IPI0076	Gene_Symb	12.4738	0.6918		325	IPI:IPI0079	Gene_Symb	22.9087	0.4831
159	IPI:IPI0021	Gene_Symb	8.3176	0.7178		326	IPI:IPI0082	Gene_Symb	18.0302	0.7047
160	IPI:IPI0002	Gene_Symb	4.9659	0.6138		327	IPI:IPI0054	Gene_Symb	8.1658	0.6855
335	IPI:IPI0045	Gene_Symb	0.2606	1.6144		334	IPI:IPI0002	Gene_Symb	1.4454	0.7656
338	IPI:IPI0047	Gene_Symb	7.2444	0.6081		558	IPI:IPI0021	Gene_Symb	87.9023	0.7178
340	IPI:IPI0079	Gene_Symb	87.9023	0.6368		563	IPI:IPI0001	Gene_Symb	0.7311	1.7539
345	IPI:IPI0003	Gene_Symb	1.0666	2.2491		572	IPI:IPI0028	Gene_Symb	10.1859	0.597
348	IPI:IPI0001	Gene_Symb	7.1121	0.5754		574	IPI:IPI0001	Gene_Symb	13.8038	0.1127
350	IPI:IPI0002	Gene_Symb	87.9023	0.6918		577	IPI:IPI0029	Gene_Symb	5.3456	0.2128
351	IPI:IPI0002	Gene_Symb	17.2187	0.3664		582	IPI:IPI0030	Gene_Symb	0.5248	1.3305
352	IPI:IPI0002	Gene_Symb	1.4191	0.4406		585	IPI:IPI0001	Gene_Symb	10.666	0.6252
356	IPI:IPI0007	Gene_Symb	0.5598	1.2589		586	IPI:IPI0003	Gene_Symb	87.9023	0.4325
365	IPI:IPI0000	Gene_Symb	5.5463	0.7656		590	IPI:IPI0000	Gene_Symb	87.9023	0.4831
367	IPI:IPI0074	Gene_Symb	99.0832	0.7943		592	IPI:IPI0021	Gene_Symb	0.3467	2.6303
368	IPI:IPI0029	Gene_Symb	0.9376	0.5808		593	IPI:IPI0022	Gene_Symb	87.9023	0.7586
371	IPI:IPI0015	Gene_Symb	86.2979	0.5346		600	IPI:IPI0029	Gene_Symb	0.263	1.3183
374	IPI:IPI0044	Gene_Symb	87.0964	0.7447		613	IPI:IPI0000	Gene_Symb	78.7046	0.4246
381	IPI:IPI0017	Gene_Symb	24.4343	0.6982		620	IPI:IPI0029	Gene_Symb	0.055	5.1523
384	IPI:IPI0002	Gene_Symb	87.9023	0.3565		632	IPI:IPI0046	Gene_Symb	14.7231	0.7943
385	IPI:IPI0001	Gene_Symb	13.3045	0.6668		637	IPI:IPI0000	Gene_Symb	87.9023	0.3404
388	IPI:IPI0001	Gene_Symb	4.8753	0.5152		638	IPI:IPI0033	Gene_Symb	8.6298	0.278
392	IPI:IPI0029	Gene_Symb	87.9023	0.4699		642	IPI:IPI0084	Gene_Symb	30.761	0.597
393	IPI:IPI0003	Gene_Symb	0.3733	1.4322		648	IPI:IPI0001	Gene_Symb	0.6792	2.3121
400	IPI:IPI0078	Gene_Symb	6.8549	0.7656		654	IPI:IPI0029	Gene_Symb	3.0479	0.4656
405	IPI:IPI0021	Gene_Symb	5.2966	0.7798		662	IPI:IPI0001	Gene_Symb	5.5463	0.7586
408	IPI:IPI0001	Gene_Symb	0.0982	18.5353		665	IPI:IPI0021	Gene_Symb	87.0964	0.2911
412	IPI:IPI0065	Gene_Symb	0.0711	1.556		667	IPI:IPI0000	Gene_Symb	87.9023	0.6855
413	IPI:IPI0001	Gene_Symb	1.9409	0.3597		683	IPI:IPI0002	Gene_Symb	1.5417	0.7943
415	IPI:IPI0017	Gene_Symb	6.9823	0.6252		695	IPI:IPI0039	Gene_Symb	12.942	0.5395
421	IPI:IPI0078	Gene_Symb	4.6132	0.787		697	IPI:IPI0087	Gene_Symb	87.9023	0.4875
425	IPI:IPI0002	Gene_Symb	99.0832	0.4966		700	IPI:IPI0037	Gene_Symb	0.0698	12.7057
428	IPI:IPI0021	Gene_Symb	2.2491	0.4207		704	IPI:IPI0003	Gene_Symb	2.208	0.5754
431	IPI:IPI0047	Gene_Symb	8.6298	0.6546		711	IPI:IPI0051	Gene_Symb	87.9023	0.7516
432	IPI:IPI0001	Gene_Symb	0.2421	5.2966		716	IPI:IPI0000	Gene_Symb	1.8707	0.7586
439	IPI:IPI0016	Gene_Symb	27.2898	0.6918		718	IPI:IPI0047	Gene_Symb	52.9663	0.6792
441	IPI:IPI0054	Gene_Symb	2.9376	0.3311		723	IPI:IPI0030	Gene_Symb	87.0964	0.7379
444	IPI:IPI0002	Gene_Symb	1.8197	0.6792		726	IPI:IPI0002	Gene_Symb	2.208	0.6138
447	IPI:IPI0088	Gene_Symb	0.0565	26.546		739	IPI:IPI0003	Gene_Symb	0.0291	3.8019
454	IPI:IPI0000	Gene_Symb	1.2706	0.6668		742	IPI:IPI0001	Gene_Symb	1.6293	0.7178
455	IPI:IPI0021	Gene_Symb	1.6749	0.3837		747	IPI:IPI0021	Gene_Symb	87.9023	0.5395
456	IPI:IPI0030	Gene_Symb	9.8175	0.3076		748	IPI:IPI0001	Gene_Symb	18.8799	0.7798
458	IPI:IPI0002	Gene_Symb	87.9023	0.5346		749	IPI:IPI0032	Gene_Symb	0.037	10.5682
461	IPI:IPI0002	Gene_Symb	29.1072	0.7516		753	IPI:IPI0002	Gene_Symb	13.6773	0.492

466	IPI:IPI0029	Gene_Symb	87.9023	0.6918		755	IPI:IPI0000	Gene_Symb	0.4699	99.0832
472	IPI:IPI0017	Gene_Symb	1.3305	0.3192		759	IPI:IPI0029	Gene_Symb	0.8551	1.4588
474	IPI:IPI0002	Gene_Symb	87.9023	0.597		765	IPI:IPI0084	Gene_Symb	0.1585	7.4473
475	IPI:IPI0021	Gene_Symb	3.3113	0.5598		769	IPI:IPI0010	Gene_Symb	0.4406	1.2023
476	IPI:IPI0002	Gene_Symb	1.5417	0.52		774	IPI:IPI0086	Gene_Symb	39.8107	0.4246
483	IPI:IPI0000	Gene_Symb	14.4544	0.7047		778	IPI:IPI0047	Gene_Symb	87.9023	0.1995
484	IPI:IPI0079	Gene_Symb	10.1859	0.4487		779	IPI:IPI0029	Gene_Symb	87.9023	0.7178
493	IPI:IPI0000	Gene_Symb	1.1588	1.803		787	IPI:IPI0079	Gene_Symb	1.9953	0.6026
494	IPI:IPI0000	Gene_Symb	3.9084	0.7656		790	IPI:IPI0084	Gene_Symb	1.5136	0.6918
506	IPI:IPI0087	Gene_Symb	0.0705	32.5087		791	IPI:IPI0002	Gene_Symb	59.7035	0.5346
514	IPI:IPI0002	Gene_Symb	0.8017	1.803		796	IPI:IPI0018	Gene_Symb	1	0.5012
518	IPI:IPI0087	Gene_Symb	87.9023	0.7943		799	IPI:IPI0002	Gene_Symb	75.8578	0.2535
519	IPI:IPI0021	Gene_Symb	0.8551	0.3802		801	IPI:IPI0002	Gene_Symb	1.406	0.6486
524	IPI:IPI0079	Gene_Symb	20.3236	0.6855		802	IPI:IPI0000	Gene_Symb	24.8886	0.5754
528	IPI:IPI0000	Gene_Symb	1.1376	2.1478		807	IPI:IPI0046	Gene_Symb	17.5388	0.3221
529	IPI:IPI0038	Gene_Symb	2.355	0.7516		810	IPI:IPI0078	Gene_Symb	14.0605	0.7727
531	IPI:IPI0079	Gene_Symb	99.0832	0.6918		814	IPI:IPI0022	Gene_Symb	87.9023	0.4742
532	IPI:IPI0021	Gene_Symb	15.8489	0.7727		817	IPI:IPI0000	Gene_Symb	35.3183	0.2228
555	IPI:IPI0001	Gene_Symb	0.0377	15.5597		818	IPI:IPI0029	Gene_Symb	0.8872	1.2706
832	IPI:IPI0079	Gene_Symb	99.0832	0.787		828	IPI:IPI0087	Gene_Symb	1.0568	1.6904
833	IPI:IPI0000	Gene_Symb	87.9023	0.6855						
838	IPI:IPI0087	Gene_Symb	6.0256	0.1837						
841	IPI:IPI0089	Gene_Symb	84.7227	0.4529						
845	IPI:IPI0015	Gene_Symb	4.7424	0.7943						

Table S4.The comparsions of immune-related signatures in the HPSCC and matched LSCC patients

Name	p.value	Adjusted.p.value
Tcm.cells	0.003	0.003
T.Cells.CD4.Memory.Resting	0.004	0.004
Mast.cells	0.015	0.015
Monocytes	0.028	0.028
Wound.Healing	0.030	0.030
Tfh.cells	0.041	0.041
TGF.beta.Response	0.043	0.043
Neutrophils	0.079	0.079
Neutrophils	0.083	0.083
Neutrophils.1	0.083	0.083
TCR.Shannon	0.096	0.096
Eosinophils	0.097	0.097
TCR.Richness	0.098	0.098
Treg.cells	0.105	0.110
Overall.immune.infiltration.score	0.115	0.110
Th17.Cells	0.127	0.130
aDC	0.134	0.130
BCR.Evenness	0.141	0.140
Dendritic.Cells.Resting	0.139	0.140
T.cell.infiltration.score	0.152	0.150
Macrophage.Regulation	0.170	0.170
Indel.Neoantigens	0.170	0.170
Eosinophils	0.171	0.170
T.Cells.CD4.Naive	0.172	0.170
Eosinophils.1	0.171	0.170
BCR.Richness	0.178	0.180
Dendritic.Cells.Activated	0.204	0.200
BCR.Shannon	0.238	0.240
Macrophages.M0	0.244	0.240
Lymphocytes	0.235	0.240
Th17.cells	0.241	0.240
DC	0.269	0.270
NK.cells	0.273	0.270
Lymphocyte.Infiltration.Signature.Score	0.301	0.300
SNV.Neoantigens	0.301	0.300
Homologous.Recombination.Defects	0.299	0.300
Macrophages	0.320	0.320
Th2.cells	0.345	0.340
T.Cells.CD8	0.350	0.350
Nonsilent.Mutation.Rate	0.375	0.370
B.cells	0.386	0.390
Proliferation	0.410	0.410

Name	p.value	Adjusted.p.value
Dendritic.Cells	0.448	0.450
Tem.cells	0.466	0.470
TCR.Evenness	0.518	0.520
iDC	0.524	0.520
B.Cells.Naive	0.534	0.530
Plasma.Cells	0.540	0.540
CD8.T.cells	0.545	0.550
Fraction.Altered	0.577	0.580
NK.Cells.Activated	0.588	0.590
CTA.Score	0.614	0.610
pDC	0.614	0.610
Tgd.cells	0.610	0.610
Silent.Mutation.Rate	0.634	0.630
T.cells	0.645	0.650
T.helper.cells	0.663	0.660
NK.CD56bright.cells	0.663	0.660
T.Cells.CD4.Memory.Activated	0.673	0.670
EMTscore	0.671	0.670
Macrophages.M2	0.686	0.690
Macrophages.M1	0.704	0.700
Mast.Cells.Activated	0.736	0.740
Cytotoxic.cells	0.744	0.740
Stromal.Fraction	0.752	0.750
Mast.Cells	0.752	0.750
Th1.cells	0.770	0.770
Mast.Cells.Resting	0.780	0.780
T.Cells.Follicular.Helper	0.817	0.820
Aneuploidy.Score	0.854	0.850
Intratumor.Heterogeneity	0.911	0.910
B.Cells.Memory	0.908	0.910
NK.Cells.Resting	0.950	0.950
Leukocyte.Fraction	0.961	0.960
IFN.gamma.Response	0.964	0.960
Number.of.Segments	0.963	0.960
NK.CD56dim.cells	0.978	0.980

R codes

```
options(stringsAsFactors = F)
mydata <- read.csv ("F:/submission/HPSCCvsLSCC/draft/manuscript/clinical 
data/SEER/LHPSCC.49171.csv", header = T)

##clean data

#rename the colnames

colnames(mydata)[1:length(colnames(mydata))]<-c('Patient.ID','Race','Gender',
                                                'Diag.year','Primary.site',
                                                'subsite','Reason.no.surgery',
                                                'COD.site.code','specific.COD',
                                                'other.COD','Survival.time',

'Survival.flag','COD.to.site.rec.KM',
                                                'Survival.status','follow.up',
                                                'Marital.status',

'Age','Histology','AJCC.stage.6th',

'AJCC.T.6th','AJCC.N.6th','AJCC.M.6th',
                                                'AJCC.stage.7th','AJCC.T.7th',
                                                'AJCC.N.7th','AJCC.M.7th'
)
#selection only 31769 cases were selected

s1<-subset(mydata,!(mydata$AJCC.stage.6th=='Blank(s)'& 
mydata$AJCC.stage.7th=='Blank(s)'))


s2<-subset(s1,!(s1$AJCC.stage.6th=='UNK Stage'& s1$AJCC.stage.7th=='UNK Stage'))

s3<-subset(s2,!((s2$AJCC.stage.6th=='UNK Stage'& s2$AJCC.stage.7th=='Blank(s)')|
                  (s2$AJCC.stage.6th=='Blank(s)'& s2$AJCC.stage.7th=='UNK 
Stage')))

s3<-subset(s3, s3$AJCC.M.6th=='M0'|s3$AJCC.M.7th=='M0') #exclude 4765 cases in 
total


# combined stage data

table(s3$AJCC.stage.6th)
table(s3$AJCC.stage.7th)
table(s3$AJCC.stage.7th[s3$AJCC.stage.6th=='UNK Stage'])
table(s3$AJCC.stage.7th[s3$AJCC.stage.6th=='IVNOS'])
table(s3$AJCC.stage.7th[s3$AJCC.stage.6th=='IVA'])
table(s3$AJCC.stage.6th[s3$AJCC.stage.7th=='Blank(s)'])
table(s3$AJCC.stage.6th[s3$AJCC.stage.7th=='UNK Stage'])

s3$AJCC.stage<-ifelse(s3$AJCC.stage.7th=='Blank(s)',s3$AJCC.stage.6th,s3$AJCC.sta
ge.7th)
```

```
s3$AJCC.T<-ifelse(s3$AJCC.stage.7th=='Blank(s)',s3$AJCC.T.6th,s3$AJCC.T.7th)

s3$AJCC.N<-ifelse(s3$AJCC.stage.7th=='Blank(s)',s3$AJCC.N.6th,s3$AJCC.N.7th)

s3$Survival.time<-as.numeric(s3$Survival.time)

table(is.na(s3$Survival.time)) # 7patients without survival data

s3$Survival.time<-ifelse(s3$Survival.time>=60,60,s3$Survival.time)

s3$Survival.status<-as.character(s3$Survival.status)

s3$Survival.status<-ifelse(s3$Survival.status=='Dead',1,ifelse(s3$Survival.status
=='Alive',0,NA))

table(s3$Survival.status)

s3$Survival.status[s3$Survival.time==60]<-0

table(s3$Survival.status)

s3$T.class<-ifelse((s3$AJCC.T%in%(c('T0','T1','T1a','T1b','T1b','T1NOS','T2'))),'
T1.2',
                   ifelse((s3$AJCC.T%in%(c('T3','T4a','T4b','T4NOS')))
                          ,'T3.4','Tx'))
s3$N.class<-ifelse((s3$AJCC.N%in%(c('N1','N2a','N2b','T1b','N2c','N2NOS','N3'))),
'N.pos',
                   ifelse((s3$AJCC.N%in%(c('NX')))
                          ,'N.x','N.neg'))

s3<-subset(s3,s3$T.class!='Tx') # delete Tx data

s3<-subset(s3,s3$N.class!='N.x') # delete Nx data
#s3 is a clean data for analysis

#####
s4<-s3
s4$AJCC.stage[s4$AJCC.stage%in%c('IVA','IVB','IVNOS')]='IV'

s4<-subset(s4,s4$AJCC.stage!='UNK Stage')

write.table(s4,file = "F:/submission/HPSCCvsLSCC/draft/manuscript/clinical
data/SEER/clean.raw.data.csv", quote = F, row.names = F, sep = ',')



s4$AJCC.T<-substr(s4$AJCC.T,1,2)


s5<-s4[,c('AJCC.T','Age','Gender','AJCC.stage','T.class','N.class','Primary.site'
,'Survival.time','Survival.status')]
library(dplyr)

s5<-na.omit(s5)%>%filter(AJCC.T!='T0')
```

```
library("survival")
library("survminer")

fit <- surv_fit(Surv(Survival.time, Survival.status)~Primary.site,
                data=s5)
F1b<-ggsurvplot_facet(fit, s5, facet.by = "AJCC.stage",
                      palette = "jco",
                      pval = TRUE, surv.median.line = "hv",
                      break.time.by=12,
                      ncol = 4,censor.size=3)

F1a<-ggsurvplot(fit, s5, risk.table = T,risk.table.pos = "in",
                #risk.table.col="strata",
                palette = "jco", pval = TRUE, surv.median.line =
"hv",break.time.by=12,censor.size=3)

table1::table1(~Primary.site|AJCC.stage,s5)

#####

library(MatchIt)

set.seed(1234)




write.table(s5,file = "F:/submission/HPSCCvsLSCC/draft/manuscript/clinical
data/SEER/cleared-LHPSCC.csv", quote = F, row.names = F, sep = ',')



s5$group<-as.logical(s5$Primary.site=="Hypopharynx")

match.it = matchit (group ~ Gender+ Age +AJCC.stage+T.class+N.class,

                    data = s5, method ="nearest", ratio =1)

plot(match.it)


a <- summary(match.it)


library(tableone)
library(knitr)
library(captioner)
library(wakefield)
library(rlang)
kable(a$nn, digits = 2, align = 'c',
      caption = 'Table 2: Sample sizes')

kable(a$sum.matched[c(1,2,4)], digits = 2, align = 'c',
```

```
      caption = 'Table 3: Summary of balance for matched data')

plot(match.it, type = 'jitter', interactive = FALSE)

tabdf.match <- match.data(match.it)[1:ncol(s5)]


summary(tabdf.match)

write.table(tabdf.match,file =
"F:/submission/HPSCCvsLSCC/draft/manuscript/clinical data/SEER/PSM-LHPSCC.csv",
quote = F, row.names = F, sep = ',')

#####

table4 <- CreateTableOne(vars = c('Gender', 'Age','T.class','N.class',
'AJCC.stage'),
                         data = tabdf.match,
                         factorVars = 'Gender',
                         strata = 'Primary.site')
table4 <- print(table4,
                printToggle = FALSE,
                noSpaces = TRUE)
kable(table4[,1:3],
      align = 'c',
      caption = 'Table 4: Comparison of matched samples')

#####
table5 <- CreateTableOne(vars = c('Gender', 'Age','T.class','N.class',
'AJCC.stage'),
                         data = s5,
                         factorVars = 'Gender',
                         strata = 'Primary.site')
table5 <- print(table5,
                printToggle = FALSE,
                noSpaces = TRUE)
kable(table5[,1:3],
      align = 'c',
      caption = 'Table5: Comparison of samples')

write.table(table4,file = "F:/submission/HPSCCvsLSCC/draft/manuscript/clinical
data/SEER/Comparison of samples.csv", quote = F, row.names = T, sep = ',')

write.table(table5,file = "F:/submission/HPSCCvsLSCC/draft/manuscript/clinical
data/SEER/Comparison of matched samples.csv", quote = F, row.names = T, sep =
',')


load("F:/TCGA/data from cbioportal/SCCHN data/SCCHN_RSEM_genes_normalized.RData")

a<-as.data.frame(a)

KMinput<-read.csv("F:/submission/HPSCCvsLSCC/batch KM input 240.100genes.csv",
sep=",",header=T, fill=T, skipNul=T, dec="-")
```

```
list.164gene<-as.character(KMinput$X240genes)

HNSCC.164<-na.omit(a[list.164gene,])

Tumor.HNSCC.164<-HNSCC.164[,substr(colnames(HNSCC.164),14,15)=='01']#癌组织的表达数据

#####

immune<-read.csv(file = 'F:/TCGA/pan-cancer/Immunity score of TCGA cancer-Cell paper.csv')



load(file = 'E:/HPV-EBV-HBV immunity in TCGA/Rdata/trimed-panCancer-TNM.RData')

clin=clin%>%select(ParticipantBarcode=bcr_patient_barcode,
                       age,
                       gender,
                       race,
                       OS,OS.time,
                       DSS,DSS.time,
                       PFI,PFI.time,
                       pathologic_T,
                       pathologic_N,
                       pathologic_M,
                       pathologic_stage,
                       Study)

HNSC.clin<-read.table("F:/TCGA/TCGA trainning/HNSCC/clinical_trimmed_data_527cancer.txt",sep="\t",header=T,check.names=F, skipNul=T,fill=TRUE)

colnames(HNSC.clin)[1]='ParticipantBarcode'

HNSC.clin$Subsite<-HNSC.clin$"Primary Tumor Site"

HNSC.clin=HNSC.clin%>%select(ParticipantBarcode,Subsite)

tmp=merge(clin,HNSC.clin,all.y = T)


{ tmp$age[is.na(tmp$age)]<-round(mean(tmp$age,na.rm = T),0) #na取平均年龄
  tmp$age<-as.numeric(tmp$age)

  #tmp[is.na(tmp)]<-'unknown'

  tmp$gender<-factor(tmp$gender,levels = c('FEMALE','MALE'), ordered = F)

  tmp$race[is.na(tmp$race)]<-'unknown'
  tmp$race[tmp$race==' [Not Evaluated]']<-'unknown'
  tmp$race<-as.factor(tmp$race)
```

```
  tmp$pathologic_T[is.na(tmp$pathologic_T)]<-'unknown'
  tmp$pathologic_T<-factor(tmp$pathologic_T,levels = c('unknown',
                                                          'T0',
                                                          'T1',
                                                          'T2',
                                                          'T3',
                                                          'T4'), ordered =
T)

  tmp$pathologic_N[is.na(tmp$pathologic_N)]<-'unknown'
  tmp$pathologic_N<-factor(tmp$pathologic_N,levels = c('unknown',
                                                          'N0',
                                                          'N1',
                                                          'N2',
                                                          'N3'
  ),ordered = T)
  tmp$pathologic_M[is.na(tmp$pathologic_M)]<-'unknown'
  tmp$pathologic_M<-factor(tmp$pathologic_M,levels = c('unknown',
                                                          'M0',
                                                          'M1'), ordered =
T)

  tmp$pathologic_stage[is.na(tmp$pathologic_stage)]<-'unknown'
  tmp$pathologic_stage<-factor(tmp$pathologic_stage,levels = c('unknown',
                                                                  'I',
                                                                  'II',
                                                                  'III',
                                                                  'IV'),
ordered = T)
  tmp$OS.time<-tmp$OS.time/30

  tmp$OS.time[tmp$OS.time>=60]<-60

  tmp$OS[tmp$OS.time==60]<-0

  tmp$DSS.time<-tmp$DSS.time/30

  tmp$DSS.time[tmp$DSS.time>=60]<-60

  tmp$DSS[tmp$DSS.time==60]<-0

  tmp$PFI.time<-tmp$PFI.time/30

  tmp$PFI.time[tmp$PFI.time>=60]<-60

  tmp$PFI[tmp$PFI.time==60]<-0

  tmp$OS<-as.factor(tmp$OS)
  tmp$DSS<-as.factor(tmp$DSS)
  tmp$PFI<-as.factor(tmp$PFI)


tmp$T.stage<-ifelse(tmp$pathologic_T%in%c('T3','T4'),'T3-4',ifelse(tmp$pathologic
_T%in%c('T1','T2'),'T1-2',NA))
```

```
  tmp$N.stage<-ifelse(tmp$pathologic_N%in%c('N3','N2','N1'),'N+',
                         ifelse(tmp$pathologic_N=='N0','N-',NA))
  tmp$M.stage<-ifelse(tmp$pathologic_M%in%c('M1'),'M1',
                         ifelse(tmp$pathologic_M%in%c('M0'),'M0',NA))
  tmp$Clinical.stage<-ifelse(tmp$pathologic_stage%in%c('I','II'),'I-II',

ifelse(tmp$pathologic_stage=='unknown',NA,'III-IV'))
  tmp$Race<-ifelse(tmp$race=='WHITE','WHITE','OTHERS')

  tmp$Age<-ifelse(tmp$age>=median(tmp$age),'Older','Younger')

}

colnames(tmp)[1]="Sample.ID"

Tumor.HNSCC.164<-as.data.frame(t(Tumor.HNSCC.164))


Tumor.HNSCC.164[,"Sample.ID"]<-substr(row.names(Tumor.HNSCC.164),1,12)

colnames(Tumor.HNSCC.164)=gsub('-','.',colnames(Tumor.HNSCC.164))

merge.164<-merge(tmp,Tumor.HNSCC.164, by = "Sample.ID")

var=colnames(Tumor.HNSCC.164)[1:(ncol(Tumor.HNSCC.164)-1)]

merge.164[,23:ncol(merge.164)]=apply(merge.164[,23:ncol(merge.164)],1,as.numeric)

merge.164.LPHSCC<-merge.164%>%filter(Subsite%in%c('Larynx','Hypopharynx'))

res.cut  <-lapply(var, function(x){k=surv_cutpoint(merge.164.LPHSCC,
                                                              time = 'OS.time',
                                                              event = "OS",
variables = x)%>%
                                    surv_categorize(.)
                                   k=k[,3]
                             })
names(res.cut)=var

ct=bind_cols(res.cut)

df=cbind(merge.164.LPHSCC[,1:22],ct)

univ_formulas <- sapply(var,function(x) {
                                   as.formula(
                                       paste('Surv(OS.time,
                                  OS==1)~', x))
                                 })

  univ_models <- lapply(univ_formulas, function(x){coxph(x, data = df)})

  # Extract data
  univ_results <- lapply(univ_models,
```

```
                                function(x){
                                  x <- summary(x)
                                  p.value<-signif(x$wald["pvalue"], digits=2)
                                  wald.test<-signif(x$wald["test"], digits=4)
                                  beta<-signif(x$coef[1], digits=2);#coeficient
beta
                                  HR <-signif(x$coef[2], digits=2);#exp(beta)
                                  HR.confint.lower <- signif(x$conf.int[,"lower
.95"], 4)
                                  HR.confint.upper <- signif(x$conf.int[,"upper
.95"],4)
                                  HR <-paste0(HR, " (", HR.confint.lower, "-",
HR.confint.upper, ")")
                                  res<-c(HR, p.value)
                                  names(res)<-c('HR(CI)', "p.value")
                                  return(res)
                                })
  res.LPHSCC.OS <- t(as.data.frame(univ_results, check.names = FALSE))

  res.LPHSCC.OS<-as.data.frame(res.LPHSCC.OS)

  res.LPHSCC.OS$p.value<-as.numeric(as.character(res.LPHSCC.OS$p.value))

  OS.univ.gene<-subset(as.data.frame(res.LPHSCC.OS),p.value<0.05) ####mRNA p<0.05


  ########multivariate cox one gene with multiple clincialpaprmeters########

  var2=rownames(OS.univ.gene)

  cc=c("gender","Age","Race","T.stage" ,"N.stage" ， "M.stage" ， "Clinical.stage")


  var3=sapply(var2,function(x) { as.formula(paste0('Surv(OS.time, OS==1)~',x,"+",
                                   paste0(cc,collapse = "+")))
                                 })

  m <- lapply(var3, function(x){

   fit=coxph(x, data = df)
   p=round(summary(fit)[["coefficients"]][1,5],3)
   HR_P=paste0('HR = ',round(summary(fit)[["conf.int"]][1,1],2),
              ' (',
              round(summary(fit)[["conf.int"]][1,3],2),
              '-',
              round(summary(fit)[["conf.int"]][1,4],2),
              ')',' ',paste0('p =
',round(summary(fit)[["coefficients"]][1,5],3)))

   data.frame(HR_p=HR_P,p.value=p)})

  m=bind_rows(m,.id = 'mRNA')

   rownames(m)=m$mRNA
```

```
   OS.multi.gene.2=m%>%filter(p.value<0.05)

   OS.vali.gene.2=intersect(OS.multi.gene.2$mRNA,rownames(OS.univ.gene))

###########################################validated data in
HNSC###########################################

var=OS.vali.gene.2


merge.164.others<-merge.164#%>%filter(!Subsite%in%c('Larynx','Hypopharynx'))


res.cut <-lapply(var, function(x){k=surv_cutpoint(merge.164.LPHSCC,
                                                             time = 'OS.time',
                                                             event = "OS",
variables = x)

c=ifelse(merge.164.others[,x]>k$cutpoint[1,1],"high","low" )


                             })
names(res.cut)=var

ct=bind_cols(res.cut)

df1=cbind(merge.164.others[,1:22],ct)


  univ_formulas <- sapply(var,function(x) {
                                 as.formula(
                                     paste('Surv(OS.time,
                                OS==1)~', x))
                               })

  univ_models <- lapply(univ_formulas, function(x){coxph(x, data = df1)})

  # Extract data
  univ_results <- lapply(univ_models,
                              function(x){
                                x <- summary(x)
                                p.value<-signif(x$wald["pvalue"], digits=2)
                                wald.test<-signif(x$wald["test"], digits=4)
                                beta<-signif(x$coef[1], digits=2);#coeficient
beta
                                HR <-signif(x$coef[2], digits=2);#exp(beta)
                                HR.confint.lower <- signif(x$conf.int[,"lower
.95"], 4)
                                HR.confint.upper <- signif(x$conf.int[,"upper
.95"],4)
                                HR <-paste0(HR, " (", HR.confint.lower, "-",
HR.confint.upper, ")")
                                res<-c(HR, p.value)
                                names(res)<-c('HR(CI)', "p.value")
```

```
                                   return(res)
                                 })
  res.LPHSCC.OS.2 <- t(as.data.frame(univ_results, check.names = FALSE))

  res.LPHSCC.OS.2<-as.data.frame(res.LPHSCC.OS.2)

  res.LPHSCC.OS.2$p.value<-as.numeric(as.character(res.LPHSCC.OS.2$p.value))

  OS.univ.other.gene<-subset(as.data.frame(res.LPHSCC.OS.2),p.value<0.05)
####mRNA p<0.05

  ########multivariate cox########

   ########multivariate cox one gene with multiple clincialpaprmeters########

  var2=rownames(OS.univ.other.gene)

  cc=c("gender","Age","Race","T.stage" ,"N.stage" , "M.stage" , "Clinical.stage")


  var3=sapply(var2,function(x) { as.formula(paste0('Surv(OS.time, OS==1)~',x,"+",
                                    paste0(cc,collapse = "+")))
                                  })

  m <- lapply(var3, function(x){

   fit=coxph(x, data = df1)
   p=round(summary(fit)[["coefficients"]][1,5],3)
   HR_P=paste0('HR = ',round(summary(fit)[["conf.int"]][1,1],2),
              ' (',
              round(summary(fit)[["conf.int"]][1,3],2),
              '-',
              round(summary(fit)[["conf.int"]][1,4],2),
              ')',' ',paste0('p =
',round(summary(fit)[["coefficients"]][1,5],3)))

   data.frame(HR_p=HR_P,p.value=p)})

  m=bind_rows(m,.id = 'mRNA')


   OS.multi.gene.2=m%>%filter(p.value<0.05)

      k=OS.multi.gene.2$mRNA

#########KM curves####
kmfigures<-lapply(k,function(x){

    kmfig<-as.formula(paste0('Surv(OS.time, OS==1)~',
                             x))
        f=ggsurvplot(surv_fit(kmfig,data=df),
                pval=TRUE,
                risk.table = T,
               censor.size=4,
```

```
                size=0.2,
                risk.table.pos='out',
                risk.table.fontsize=2.5,
                risk.table.y.text=F,
                legend="none",
                pval.size=2.5,
                break.time.by=12,
                palette='npg',
                tables.col = "strata",
                xlab = "Time (months)",
                 title = paste0(LETTERS[grep(x,k)],"."," ",x," ( OS)"),
                 surv.median.line = "hv")
  f$plot<-f$plot + theme(plot.title = element_text(hjust = 0.5, face =
"bold",size=8))
  f=f+theme_survminer(base_size = 8,
                      font.main = c(8, "bold", "black"),
                      font.submain = c(8, "bold", "black"),
                      font.caption = c(8, "plain", "black"),
                      font.x = c(8, "bold", "black"),
                      font.y = c(8, "bold", "black"),
                      font.tickslab = c(8, "plain", "black"),
                      font.legend = c(8, "plain", "black"),
                      legend = "none"
                                        )
    return(f)
})
gsurvlist<-arrange_ggsurvplots(kmfigures[1:9],
                                ncol=3, nrow=3,print = T)  ###

ggsave(gsurvlist,filename = './Figures/Figure5_OS_LHPSCC.pdf',width = 7.5,height
= 10,
         units = 'in',dpi = 1200)

kmfigures2<-lapply(k,function(x){

    kmfig<-as.formula(paste0('Surv(OS.time, OS==1)~',
                             x))
        f=ggsurvplot(surv_fit(kmfig,data=df1),
                 pval=TRUE,
                 risk.table = T,
                censor.size=4,
                size=0.2,
                risk.table.pos='out',
                risk.table.fontsize=2.5,
                risk.table.y.text=F,
                legend="none",
                pval.size=2.5,
                break.time.by=12,
                palette='npg',
                tables.col = "strata",
                xlab = "Time (months)",
                 title = paste0(LETTERS[grep(x,k)],"."," ",x," ( OS)"),
                 surv.median.line = "hv")
  f$plot<-f$plot + theme(plot.title = element_text(hjust = 0.5, face =
```

```
"bold",size=8))
  f=f+theme_survminer(base_size = 8,
                      font.main = c(8, "bold", "black"),
                      font.submain = c(8, "bold", "black"),
                      font.caption = c(8, "plain", "black"),
                      font.x = c(8, "bold", "black"),
                      font.y = c(8, "bold", "black"),
                      font.tickslab = c(8, "plain", "black"),
                      font.legend = c(8, "plain", "black"),
                      legend = "none"
                                         )
    return(f)
})
gsurvlist2<-arrange_ggsurvplots(kmfigures2[1:9],
                                ncol=3, nrow=3,print = T)  ###

ggsave(gsurvlist2,filename = './Figures/Figure6_OS_HNC.pdf',width = 7.5,height =
10,
         units = 'in',dpi = 1200)




clin.LHPSCC<-subset(clin_data[,c(2,14,31:34,54,61)],clin_data$Primary.Tumor.Site=
='Hypopharynx'|clin_data$Primary.Tumor.Site=='Larynx')

row.names(clin.LHPSCC)<-clin.LHPSCC[,1]

library(ggalluvial)
library(foreach)
library(dplyr)
library(ggpubr)
library(ggplotify)
library(cowplot)
library(readxl)

library(MatchIt) #####NA in the input dataframe isn't allowed 不允许输入数据中存
在NA.
library(tableone)
library(knitr)
library(captioner)
library(wakefield)
library(rlang)
library(reshape2)
options(stringsAsFactors = F)

set.seed(1234)###

HNSC.clin<-read.table("F:/TCGA/TCGA
trainning/HNSCC/clinical_trimmed_data_527cancer.txt",sep="\t",header=T,check.name
s=F, skipNul=T,fill=TRUE)

#table(HNSC.clin$"Primary Tumor Site" )
```

```
HNSC.clin$Subsite.1<-recode(HNSC.clin$"Primary Tumor Site",
                            'Alveolar Ridge'='Oral.cavity',
                            'Base of tongue'='Oropharynx',
                            'Buccal Mucosa'='Oral.cavity',
                            'Floor of mouth'='Oral.cavity',
                            'Hard Palate'='Oral.cavity',
                            'Lip'='Oral.cavity',
                            'Oral Cavity'='Oral.cavity',
                            'Oral Tongue'='Oral.cavity',
                            'Tonsil'='Oropharynx'
)

colnames(HNSC.clin)[1]<-colnames(clin.data)[1]

colnames(HNSC.clin)[41]<-'Histologic.type'

colnames(HNSC.clin)[40]<-'Histologic.grade'


dl<-subset(HNSC.clin,HNSC.clin$'Primary Tumor Site'%in%c('Hypopharynx','Larynx'))

dl[is.na(dl)]<-'unknown'
dl<-na.omit(dl)

dl$group<-as.logical(dl$Subsite.1=="Hypopharynx")
colnames(dl)<-colnames(dl)%>%gsub(' ','.',.)
match.it = matchit (group ~ Person.Gender+ Diagnosis.Age +cStage,

                    data = dl, method ="nearest", ratio =1)
matched.ID<-match.it[["match.matrix"]]

match.out<-match.data(match.it, group="all", distance = "distance",weights =
"weights", subclass = "subclass")

matched.HPSCC<-row.names(matched.ID)

matched.LSCC<-matched.ID[,1]

matched.HNSC<-dl[c(matched.HPSCC,matched.LSCC),]%>%select(ParticipantBarcode=1)%>
%mutate(site=c(rep('Hypopharynx',10),rep('Larynx',10)))


##################
cibersort.i<-read.csv(file = 'F:/TCGA/pan-cancer/Resource/immunity of
cancer/Cellular Fraction Estimates/CIBERSORT immune fractions
-TCGA.Kallisto.fullIDs.cibersort.relative.tsv',
                      sep =
'\t')%>%select(-c(2,25:27))%>%filter(substr(SampleID,14,15)=='01')


cibersort.i$SampleID<-substr(cibersort.i$SampleID,1,12)%>%gsub('\\.','-',.)

colnames(cibersort.i)[1]<-"ParticipantBarcode"
```

```
colnames(cibersort.i)[which(colnames(cibersort.i)=='T.cells.regulatory..Tregs.')]
='T.cells.regulatory.Tregs'


meta<-merge(matched.HNSC,cibersort.i)

######HPV+OP,HPV+Non-OP,HPV-HNSC,HPV-CESC,HPV+CESC######

  t<-meta%>%split(.$site)
tmp<-list()

for (i in names(t)) {tmp[[i]]=t[[i]]%>%select(3:ncol(.))}


Mean.dat.i<-sapply(tmp, colMeans)


Mean.dat.i<-as.data.frame(Mean.dat.i)

meta1<-t(Mean.dat.i)

meta1<-as.data.frame(meta1)
meta1$Subsite<-rownames(meta1)
meta1<-meta1%>%melt('Subsite',value.name = 'Proportion')
colnames(meta1)[2]<-'Immune.cell'
table(meta1$Subsite)
meta1$Subsite<-factor(meta1$Subsite,levels = c('Hypopharynx','Larynx'))



########Fig1a. Global immune cell across HPV-driven and tissue source
tumors######

ggplot(meta1, aes(x = Subsite, fill = Immune.cell, stratum = Immune.cell,
                  alluvium = Immune.cell, y = Proportion, label = Immune.cell)) +
  theme_classic() +
  theme(axis.title.x = element_blank())+
  geom_flow() +
  geom_stratum()+
  #geom_fit_text(aes(label =Immune.cell)) +
  labs(x='Subsite',y='Proportion of Tumor infilrating cells')+
  ggsave(filename = 'F:/submission/HPSCCvsLSCC/JCI-insight/The global immunity
cell across LHPSCC.cibersort.png',
         width = 11,height = 6,dpi = 1200)


###################
load(file = 'F:/TCGA/pan-cancer/Pan-cancer/primary.solid.tumor.mRNA.expr.RData')
##mRNA expression data ps.data

ps.data$ParticipantBarcode<-row.names(ps.data)

mRNA<-ps.data%>%filter(ParticipantBarcode%in%matched.HNSC$ParticipantBarcode)%>%s
```

```
elect(-c("TCGA Participant Barcode",

        "ParticipantBarcode"))
rm(ps.data)

umap.total.2<-umap(as.matrix(mRNA), n_neighbors = 50)

plot2<-umap.total.2%>%
  mutate(vir=matched.HNSC$site)%>%
  ggplot(aes(UMAP1, UMAP2, color = vir)) +
  geom_point()

plot2
######################
ICB.marker<-read_excel("../../immune checkpoint biomarkers.xlsx", sheet =
'genelist',col_names = T)

ICB.exp<-mRNA%>%select(ICB.marker$ICB.makers)

######################

######
library(ComplexHeatmap)


  Subsite=matched.HNSC$site
  ha = HeatmapAnnotation(Subsite=Subsite
                        )
  ICB.exp%>%
    t()%>%as.matrix()%>%
    apply(1,scale)%>%t()%>%
    Heatmap(name = 'ICB',
            clustering_distance_columns = function(x, y) 1 - cor(x, y),
            cluster_columns = cluster_within_group(., Subsite),
            top_annotation =ha,
            show_column_names = F,
            rect_gp = gpar(col = "white", lwd = 2)
    )

##################

  immune<-read.csv(file = 'F:/TCGA/pan-cancer/Immunity score of TCGA cancer-Cell
paper.csv')

  #immune[is.na(immune)]<-0

  colnames(immune)[1]<-"ParticipantBarcode"

  matched.LHPSCC.cellpaper<-merge(matched.HNSC,immune,all=F)

  ####cell paper
  #TGF.beta.Response
  {TGF.beta.Response<-ggpaired(matched.LHPSCC.cellpaper, x="site",
y="TGF.beta.Response",
```

```
                                color="site", line.color="gray",
                                line.size=0.4, palette = "jco",
                                title='Estimation of TGF-β Response',
                                xlab = FALSE,
                                ylab = 'Score',
                                legend = "none") +
      stat_compare_means(paired = TRUE, method = 't.test',label.x = 0.6)+
      theme(plot.title = element_text(hjust = 0.5, face = "bold",size=10))
  }

  #Wound.Healing
  {Wound.Healing<-ggpaired(matched.LHPSCC.cellpaper, x="site", y="Wound.Healing",
                            color="site", line.color="gray",
                            line.size=0.4, palette = "jco",
                            title='Estimation of Wound Healing Capability',
                            xlab = FALSE,
                            ylab = 'Score',
                            legend = "none") +
      stat_compare_means(paired = TRUE, method = 't.test',label.x = 0.55)+
      theme(plot.title = element_text(hjust = 0.5, face = "bold",size=10))
  }
  # Monocytes
  { Monocytes<-ggpaired(matched.LHPSCC.cellpaper, x="site", y="Monocytes",
                         color="site", line.color="gray",
                         line.size=0.4, palette = "jco",
                         title='Estimation of Monocytes',
                         xlab = FALSE,
                         ylab = 'Score',
                         legend = "none") +
      stat_compare_means(paired = TRUE, method = 't.test',label.x = 0.6)+
      theme(plot.title = element_text(hjust = 0.5, face = "bold",size=10))
  }

  #T.Cells.CD4.Memory.Resting
  {T.Cells.CD4.Memory.Resting<-ggpaired(matched.LHPSCC.cellpaper, x="site",
y="T.Cells.CD4.Memory.Resting",
                                        color="site", line.color="gray",
                                        line.size=0.4, palette = "jco",
                                        title='Estimation of CD4+ T memory
resting cells',
                                        xlab = FALSE,
                                        ylab = 'Score',
                                        legend = "none") +
      stat_compare_means(paired = TRUE, method = 't.test',label.x = 0.6)+
      theme(plot.title = element_text(hjust = 0.5, face = "bold",size=10))
  }
  ####MKSCC
  load(file = "f:/TCGA/immune score from MKSCC/immunescore with EMT
status.RData")

  c$'Sample.ID'<-row.names(c)

  colnames(c)[26]<-"T.cell.infiltration.score"
```

```
colnames(c)[27]<-"Overall.immune.infiltration.score"

colnames(c)<-gsub('_','.',colnames(c))
c$ParticipantBarcode=substr(rownames(c),1,12)
matched.MKSCC<-merge(matched.HNSC,c,all=F)
#Tcm.cells
{Tcm.cells<-ggpaired(matched.MKSCC, x="site", y="Tcm.cells",
                     color="site", line.color="gray",
                     line.size=0.4, palette = "jco",
                     title='Estimation of Tcm cells',
                     xlab = FALSE,
                     ylab = 'Score',
                     legend = "none") +
    stat_compare_means(paired = TRUE, method = 't.test',label.x = 0.6)+
    theme(plot.title = element_text(hjust = 0.5, face = "bold",size=10))
}
#Tfh.cells
{Tfh.cells<-ggpaired(matched.MKSCC, x="site", y="Tfh.cells",
                     color="site", line.color="gray",
                     line.size=0.4, palette = "jco",
                     title='Estimation of Tfh cells',
                     xlab = FALSE,
                     ylab = 'Score',
                     legend = "none") +
    stat_compare_means(paired = TRUE, method = 't.test',label.x = 0.6)+
    theme(plot.title = element_text(hjust = 0.5, face = "bold",size=10))
}

#Mast.cells
{Mast.cells<-ggpaired(matched.MKSCC, x="site", y="Mast.cells",
                      color="site", line.color="gray",
                      line.size=0.4, palette = "jco",
                      title='Estimation of Mast cells',
                      xlab = FALSE,
                      ylab = 'Score',
                      legend = "none") +
    stat_compare_means(paired = TRUE, method = 't.test',label.x = 0.6,label =
)+
    theme(plot.title = element_text(hjust = 0.5, face = "bold",size=10))
}

#####arrange

P1=ggarrange(Tcm.cells, Wound.Healing, Tfh.cells,Mast.cells,
          TGF.beta.Response,T.Cells.CD4.Memory.Resting,
          Monocytes,Mast.cells,
          ncol = 4, nrow = 2,labels = c(LETTERS[3:10]))

Fig1<-ggdraw() +
  draw_plot( P1)+
  ggsave(filename='F:/submission/HPSCCvsLSCC/JCI-insight/immunecell.png',
         width = 18,height = 9,
         dpi = 1200)
```
